# Phylogeography of the widely distributed John Dory ( *Zeus faber* , Actinopterygii: Zeiformes) reaffirms the prevalence of at least two deeply divergent clades

**DOI:** 10.1111/jfb.70245

**Published:** 2025-10-06

**Authors:** João Tadeu Fontes, Kenza Mokhtar‐Jamaï, Zakariya Nchioua, Jean‐Dominique Durand, Monica Landi, João Meira, Luís Machado, Ayoub Baali, Ignacio Sobrino, Iça Barri, Emmanuel Kouamé, Béatrice Abouo Adepo‐Gourène, Mamadou Diop, Néné Gallé Kidé, Austin Saye Wehye, Zacharie Sohou, Miguel Carneiro, Rogélia Martins, Pedro Soares, Filipe Oliveira Costa

**Affiliations:** ^1^ Centre of Molecular and Environmental Biology (CBMA)/ARNET‐Aquatic Research Network, Department of Biology University of Minho Braga Portugal; ^2^ Institute of Science and Innovation for Bio‐Sustainability (IB‐S), University of Minho Braga Portugal; ^3^ Institut National de Recherche Halieutique (INRH) Centre Régional d'Agadir, Laboratoire de Génétique des Populations Halieutiques Agadir Morocco; ^4^ MARBEC, Univ. Montpellier, IRD, CNRS Montpellier France; ^5^ West African Marine Fish DNA Barcoding Network (IRN WAMBA‐Net) Montpellier France; ^6^ Italian Ministry of Education and Merit, Istituto Comprensivo Baccio da Montelupo Montelupo Fiorentino Italy; ^7^ Institut National de Recherche Halieutique (INRH) Centre Régional d'Agadir, Laboratoire de Prospections Démersales Agadir Morocco; ^8^ Centro Oceanográfico de Cádiz Instituto Español de Oceanografía – Spanish National Research Council (IEO‐CSIC) Cádiz Spain; ^9^ Instituto Nacional de Investigação das Pescas e Oceanografia (INIPO) Bissau Guinea‐Bissau; ^10^ Department of Natural Sciences Université Nangui Abrogoua (UNA) Abidjan Côte d'Ivoire; ^11^ Direction des Aires Marines Communautaires Protégées (DAMCP) Dakar Senegal; ^12^ Institut Superieur des Sciences de la Mer (ISSM) Nouadhibou Mauritania; ^13^ Department of Research and Statistics, National Fisheries and Aquaculture Authority (NaFAA) Montserrado Liberia; ^14^ Institut de Recherches Halieutiques et Océanologiques du Bénin (IRHOB) & Department of Zoology University of Abomey‐Calavi Abomey‐Calavi Benin; ^15^ Modelling and Management Fishery Resources Division (DIV‐RP), Instituto Português do Mar e da Atmosfera (IPMA) Lisbon Portugal

**Keywords:** DNA barcoding, Eastern Atlantic, evolutionary history, phylogeographic break

## Abstract

The John Dory *Zeus faber* is a commercially exploited demersal fish species with a known distribution ranging from the Northeast Atlantic to parts of the Indian and Pacific oceans. A previous genetic survey using cytochrome c oxidase subunit I (COI) DNA barcodes suggested the presence of two geographically segregated taxonomic units within *Z. faber*. We revisit this hypothesis by expanding the number and geographic coverage of DNA barcodes, addressing a major data gap along parts of the Atlantic coast of Africa and conducting a comprehensive phylogeographic analysis. Our findings consolidated the existence of two highly divergent mitochondrial clades, Clade A and Clade B (mean K2P distance: 7.4%), with the transition zone between them located along the Atlantic coast of Morocco. Clade A exhibited no phylogeographic structure, with haplotypes shared between Northeast Atlantic and Mediterranean populations. Conversely, four geographically structured subclades (mean K2P distance: 0.9%) were detected within Clade B, extending south and eastward from Morocco to Japan and New Zealand. Historical demographic events driving allopatric divergence, along with oceanographic and environmental factors, likely shaped the current geographic distribution of the two clades. These findings not only prompt the need to re‐evaluate the taxonomic status of *Z. faber* but also highlight the probable existence of multiple evolutionarily significant units (ESUs) that must be considered in the scope of stock assessment, fisheries management and conservation purposes.

## 1 INTRODUCTION

Marine environments encompass vast geographic ranges and diverse ecological regions, providing opportunities to explore unique patterns of genetic divergence and phylogeographic structure within species (Avise et al., 2016; Dalongeville et al., 2022; Grant & Bowen, 1998; Palumbi, 1997). Despite being highly interconnected, marine ecosystems are influenced by a combination of historical, oceanographic and ecological factors that can promote geographic isolation and, therefore, shape complex patterns of intraspecific genetic divergence and speciation (Martins et al., 2022; Salmenkova, 2011). Historically, marine fishes were considered capable of maintaining genetic connectivity across large distribution ranges due to their high dispersal abilities, particularly during the pelagic larval phase. However, phylogeographic studies and DNA barcoding surveys have increasingly revealed cryptic diversity in species once thought to have broad distribution ranges (Arroyave et al., 2019; Guimarães et al., 2022; Jamaludin et al., 2020). In recent years, several species complexes have been unravelled, including, for instance, *Omobranchus punctatus* (Valenciennes 1836), *Lampris guttatus* (Brünnich 1788) and *Mugil cephalus* L. 1758 (Cabezas et al., 2022; Crosetti et al., 1993; Hyde et al., 2014; Whitfield et al., 2012). Indeed, a notable study compared species occurring on opposite sides of the Indian Ocean and found that one‐third of these species may individually represent two distinct taxonomic units (Zemlak et al., 2009).

Marine fishes, due to their wide distributions and exposure to diverse ecological pressures, are well‐suited models for the study of marine phylogeography (Avise et al., 2000; Grant & Bowen, 1998; Kottillil et al., 2023). Phylogeographic analysis helps distinguish evolutionarily significant units (ESUs), which may represent reproductively isolated populations. Identifying these units can ensure that conservation and fisheries management efforts, such as setting appropriate fishing restrictions or protecting vulnerable populations, are accurately designed (Ferrette et al., 2021; Hüne et al., 2021; Ollé‐Vilanova et al., 2024). Furthermore, identifying potential phylogeographic discontinuities in marine fishes helps understand how past events could have shaped current distributions (Arroyave et al., 2019; Shen et al., 2011). Particularly, the cytochrome c oxidase subunit I (COI) DNA barcode region has been extensively used to identify marine fish species, particularly in cases where taxonomic ambiguities required resolution (Bingpeng et al., 2018; Chang et al., 2017; Landi et al., 2014; Liu et al., 2022; Oliveira et al., 2016). It has also proven to be an effective marker for delimiting divergent genetic lineages through phylogeographic analysis and detecting hidden diversity in fishes (Arroyave et al., 2019; Cabezas et al., 2022; Guimarães et al., 2022; Nneji et al., 2020; Vilasboa et al., 2022).

The John Dory *Zeus faber* L. 1758 is a commercially important demersal marine fish species, prominently for human consumption (Giarratana et al., 2018; Iwamoto, 2015). It is typically captured as by‐catch during bottom trawls and occasionally targeted seasonally (Dunn, 2001; Ismen et al., 2013; Kim et al., 2020). Despite reports of heavy exploitation (Gascuel et al., 2007; Vrgoč et al., 2006), the International Union for Conservation of Nature (IUCN) Red List of Threatened Species categorizes the species as ‘data deficient’ as of 13 January 2025 (Iwamoto, 2015). This species has a vast geographic distribution spanning European, African, Asian and Australian coastal regions (Janssen, 1979; Ward et al., 2008; Yoneda et al., 2002) separated by several well‐known biogeographic barriers. The pelagic larval duration of *Z. faber* remains unknown, limiting our ability to predict its dispersal potential. Pelagic larval duration is a key determinant of genetic structure in marine species, with longer durations usually promoting gene flow between distant populations (Treml et al., 2012). Interestingly, Ward et al. (2008) observed deep genetic divergence in the COI DNA barcode region for *Z. faber*. This divergence was observed between specimens collected in Australia and New Zealand versus those collected in the Northeast Atlantic Ocean and Mediterranean Sea. These two clades currently correspond to two distinct barcode index numbers (BINs; Ratnasingham & Hebert, 2013) in the Barcode of Life Data Systems (BOLD; Ratnasingham et al., 2024; accessed 13 January 2025), which are generally considered distinct molecular operational taxonomic units (MOTUs). Nonetheless, the distribution of the two clades, particularly along the Atlantic coast of Africa, has remained largely unexplored. Moreover, since the study by Ward et al. (2008), additional DNA barcodes for this species have been added to public databases, enriching the dataset available for phylogeographic analysis.

In this study, we investigated the genetic divergence and evolutionary history of *Z. faber* by expanding the geographic coverage of the study by Ward et al. (2008). We addressed the large barcode data gap along the Atlantic coast of Africa, namely Angola, Benin, Côte d'Ivoire, Liberia, Guinea‐Bissau, Senegal, Mauritania and Morocco. By generating new COI sequences from specimens collected in the coastal waters of these countries and integrating them with publicly available data, we aimed to perform a comprehensive phylogeographic analysis to deepen our understanding of this species' diversity and evolutionary history. Our goal was to elucidate the geographic span of the two previously detected deeply divergent clades identified by Ward et al. (2008), map the transition region and screen for genetic structure. Such information is relevant for unravelling *Z. faber*'s evolutionary history, as well as for accurate and reliable stock assessments and fisheries management. Additionally, the findings of this study will enhance our understanding of the evolutionary history of fish species in the Eastern Atlantic Ocean, a region where phylogeographic breaks have been reported (Bargelloni et al., 2003; Caballero‐Huertas et al., 2022; Catarino et al., 2017; Chahdi Ouazzani et al., 2017; Cunha et al., 2022).

## 2 MATERIALS AND METHODS

### 2.1 Specimen collection and sequence generation

A total of 100 *Z. faber* specimens were collected from the coastal waters of Angola, Benin, Côte d'Ivoire, Guinea‐Bissau, Liberia, Mauritania, Morocco, Portugal and Senegal. All specimens were identified morphologically, and a small portion of skeletal muscle tissue (~0.5–1 cm^3^) was extracted from each for genetic analysis. Collection metadata, GenBank accession numbers and BOLD process IDs are provided in Table S1. A ~ 652‐bp fragment of the 5′ end of the COI gene (COI‐5P), corresponding to the DNA barcode region (Ivanova et al., 2007; Ward et al., 2005), was amplified and Sanger sequenced following standard DNA barcoding protocols. Laboratory‐specific procedures used for sequence generation are detailed in Table S2.

All specimens used in this study were obtained in accordance with applicable national and international laws, including access and benefit‐sharing (ABS) frameworks where relevant. Eight samples were sourced from artisanal fish landings in Côte d'Ivoire, Mauritania and Senegal, from publicly accessible markets where no formal collection permits were required under local regulations. The remaining 92 specimens were collected during authorized research surveys conducted by institutional partners in Angola, Benin, Liberia, Guinea‐Bissau, Morocco and Portugal. Tissue samples were taken post‐mortem from fish already captured through legal fishing activities. No live animals were handled or killed for the purposes of this study.

### 2.2 Dataset compilation

**FIGURE 1:**
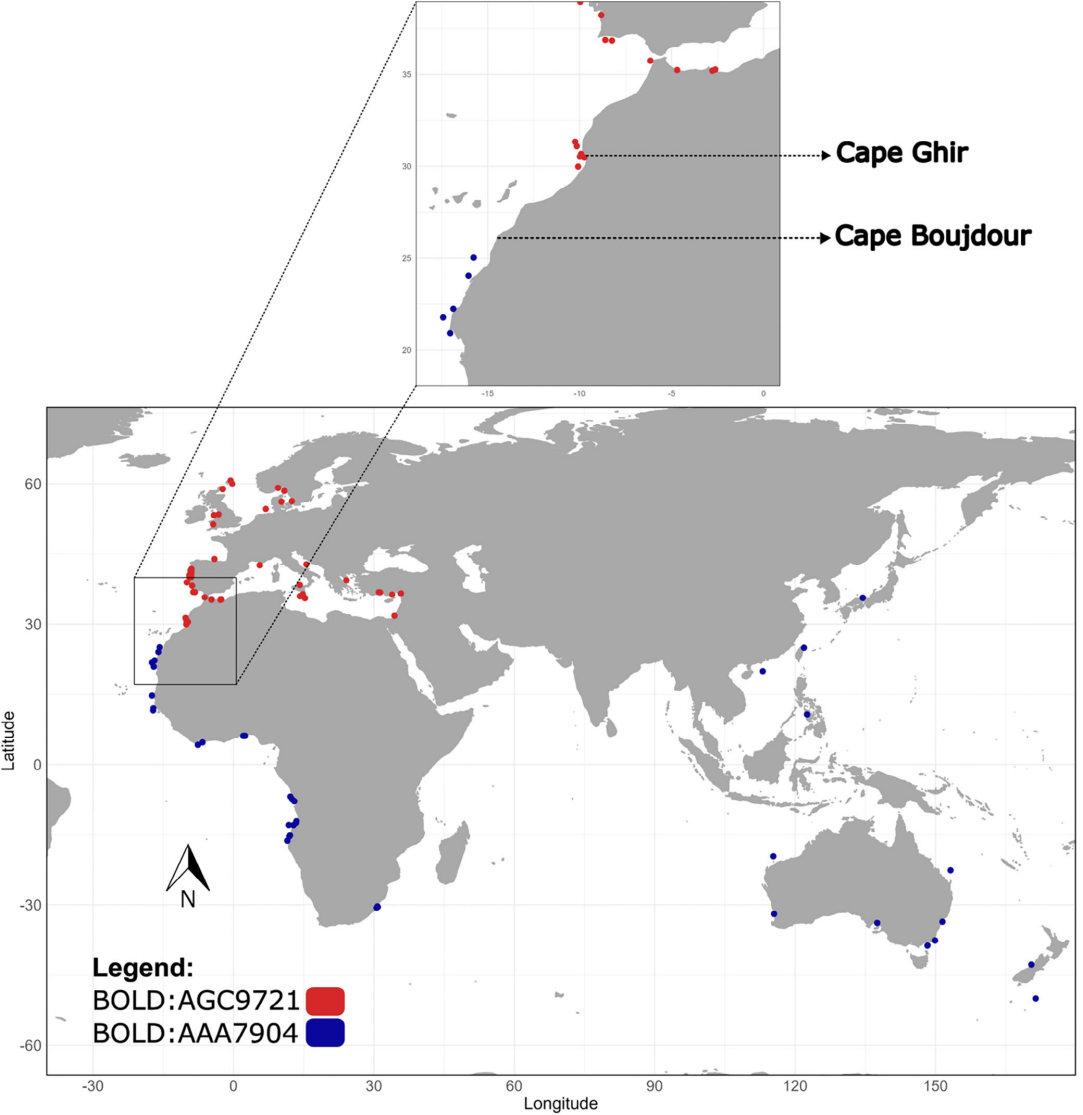
Sampling locations for the *Zeus faber* specimens, including the newly generated sequences and records mined from BOLD. Point colours in the legend correspond to the barcode index number (BIN) assigned to each record in the BOLD database.

The identity of the generated sequences was confirmed using the BOLD identification tool, and the absence of stop codons in all sequences was confirmed within the BOLD workbench (Ratnasingham et al., 2024). Subsequently, the dataset was enriched with 122 *Z. faber* sequences downloaded from BOLD. During the preliminary analysis, 21 of the mined sequences introduced a disproportionate number of reticulations (i.e., loops or conflicting connections) in the reduced‐median network (Bandelt et al., 1995). Such patterns are often caused by artefactual mutations occurring inconsistently across sequences, which create multiple, equally plausible evolutionary pathways. This interpretation is consistent with the unusually high number of mutations observed in these sequences, raising concerns about their quality and suggesting potential sequencing or base‐calling errors. As access to the sequencing trace files was unavailable, preventing further quality verification, these sequences were excluded from subsequent analyses. The sampling locations for all *Z. faber* records utilized in this study with available geographic information are displayed in Figure 1. World map data were obtained using the *maps* package version 3.4.1 (Becker et al., 2022), originally retrieved from the Natural Earth data project (1:50 m, version 2.0, 2015, https://www.naturalearthdata.com). For specimens without geographic co‐ordinates, approximate locations were assigned based on the most‐detailed available information, such as seas, regions or countries; these cases are provided in Table S3. Additional DNA barcode sequences belonging to the family Zeidae, specifically *Zeus capensis* Valenciennes 1835, *Zenopsis conchifer* (Lowe 1852) and *Zenopsis nebulosa* (Temminck & Schlegel 1845), were also retrieved from BOLD to be incorporated in the analysis as out‐groups. The total number of mined *Z. faber* DNA barcodes included in this study was 101, with 43 additional sequences from the out‐groups. The BOLD process IDs and corresponding species names for the mined DNA barcodes used in this study are provided in Table S3.

### 2.3 Data analysis

All sequences were aligned using ClustalW (Thompson et al., 1994) with default parameters in Mega 11 (Tamura et al., 2021). To reconstruct the phylogeny and analyse the evolutionary relationship between haplotypes, polymorphisms were identified using mtDNA‐GeneSyn (Pereira et al., 2009), and a haplotype network was constructed using the reduced‐median algorithm (Bandelt et al., 1995) in Network version 10.2.0.0 (www.fluxus-engineering.com). The multiple sequence alignment included sequences varying in length, resulting in gaps at the extremities. To avoid losing valuable genetic information and geographic coverage, we used a reconstruction approach based on the definition of the most parsimonious missing segments of DNA for each sequence. Missing segments for shorter sequences were reconstructed using shared or most closely related haplotypes based on the phylogenetic reconstruction provided by the reduced‐median network. Overall, 2.6% of the alignment matrix (i.e., calculated as 201 sequences × 620 positions) was reconstructed, with 9.4% of the reconstructed positions being polymorphic. This approach resulted in a multiple sequence alignment of 620 positions used in downstream analyses. Supplementary analyses were conducted by trimming the original non‐reconstructed alignment to match the shortest sequence, resulting in a multiple sequence alignment of 408 bp. The results showed that the key trends and relative differences were unchanged compared to the reconstructed alignment and did not alter the interpretation or discussion of the results. These supplementary results are detailed in Tables S4–S8.

To measure genetic divergence, a Kimura‐2‐Parameter (K2P; Kimura, 1980) distance matrix was computed in Mega 11. Polymorphic (segregating) sites (S), and parsimony‐informative sites (PIS) were determined using the *ape* R package version 5.5 (Paradis & Schliep, 2019), whereas the number of haplotypes (H), haplotype diversity (Hd) and nucleotide diversity (*π*) were calculated using the *pegas* R package version 1.1 (Paradis, 2010). These analyses were performed overall for the entire dataset, for each main clade identified, and separately for different geographic groups. The geographic groups were defined as follows: (1) the Northeast Atlantic, which included samples from Europe, as well as the Northern and Central Atlantic regions of Morocco (bounded north by Cape Spartel and south by Cape Boujdour); (2) the Mediterranean Sea, comprising samples from its western, central and eastern regions; (3) Atlantic Africa, which comprised samples from the western coast of Africa, including only the Southern Atlantic region of Morocco (bounded north by Cape Boujdour and south by Cape Blanc); (4) South Africa, encompassing samples from the Indian Ocean; (5) Australia (from the Indian and Pacific Ocean regions) and New Zealand; and (6) Asia, comprising samples from Japan, China, Taiwan and the Philippines. To calculate genetic diversity metrics, the dataset for each geographic group was normalized by subsampling to match the second smallest sample size (Asia, *n* = 12). The smallest group (South Africa, *n* = 4) was excluded because it was too small to yield robust estimates and contained only a single haplotype, preventing the calculation of haplotype and nucleotide diversity. Subsampling was repeated 10,000 times without replacement, and mean values for each genetic diversity metric were calculated. Tajima's D was calculated for each geographic group to test deviations from neutrality, using the *pegas* R package. An analysis of molecular variance (AMOVA) was conducted using Arlequin version 3.5.2.2 (Excoffier et al., 1992, 2007) with 100,000 permutations, utilizing K2P distances. The analysis was performed to assess the genetic structure of *Z. faber* at two hierarchical levels: among clades and among geographic groups. For analysis using the previously defined geographic groups, we excluded sequences without geographic origin information, as well as one sequence labelled as being collected in the Mid‐Atlantic Bight (see Supplementary Text S1 for details).

A Bayesian phylogenetic tree was constructed using BEAST version 1.10.4 (Suchard et al., 2018), employing the HKY + I + G evolutionary model, which was selected based on the Bayesian information criterion (BIC) analysis performed using jModeltest version 2.1.10 (Darriba et al., 2012). The Bayesian analysis ran for 100 million Markov chain Monte‐Carlo (MCMC) iterations, sampling every 10,000th generation. A mutation rate of 1.2% per million years (with a standard error of 10%) was set as a prior based on previous estimations for marine bony fishes (Bermingham et al., 1997; Hüne et al., 2021; Reece et al., 2010). Additionally, a fossil calibration prior was applied to the crown node of the Zeidae family, using an estimated age of 32.02 million years as described in the phylogeny by Rabosky et al. (2018); https://fishtreeoflife.org/taxonomy/family/Zeidae/. The Bayesian analysis estimated a mutation rate of 9.94 × 10^−9^ substitutions per site per year (0.994% per million years), with a 95% credible interval (CI) of 7.87–12.0 × 10^−9^ substitutions per site per year. The coalescence time for *Z. faber* was estimated at 4.81 million years (95% CI: 3.89–5.84 million years). For the Zeidae family, the coalescence time was estimated at 25.7 million years (95% CI: 18.6–33.0 million years). To estimate population expansions associated with each major clade, we calculated two Bayesian Skyline Plots (BSPs) in BEAST using the previously obtained substitution rate (9.94 × 10^−9^ substitutions per site per year). BSPs were visualized using Tracer version 1.7.2 (Rambaut et al., 2018). For MOTU delimitation, two methods were used. The Refined Single Linkage algorithm (RESL) was applied as implemented in the BOLD databased through the BIN system (Ratnasingham & Hebert, 2013), and the automatic Assemble Species by Automatic Partitioning (ASAP; Puillandre et al., 2021) was employed on https://bioinfo.mnhn.fr/abi/public/abgd/abgdweb.html with default parameters using K2P distances.

## 3 RESULTS

**FIGURE 2:**
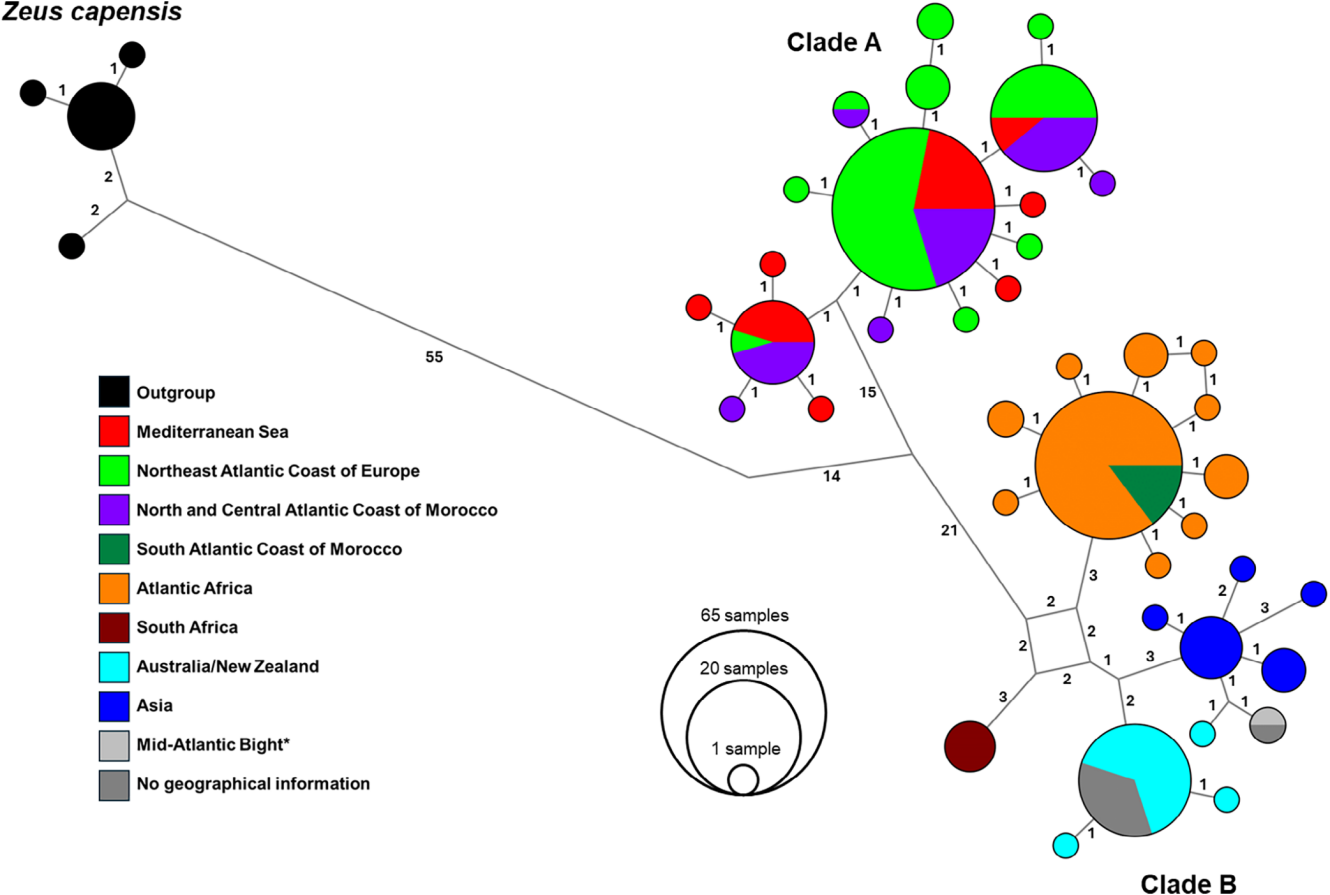
Reduced‐median haplotype network for *Zeus faber* based on 620 bp of the COI barcode region. The size of the circles is proportional to the number of individuals sharing the haplotype. The numbers on the edges indicate the number of mutational steps connecting haplotypes and median vectors. An asterisk (*) indicates that additional information regarding the sample annotated as having been collected in the Mid‐Atlantic Bight is provided in Data S1.

**FIGURE 3:**
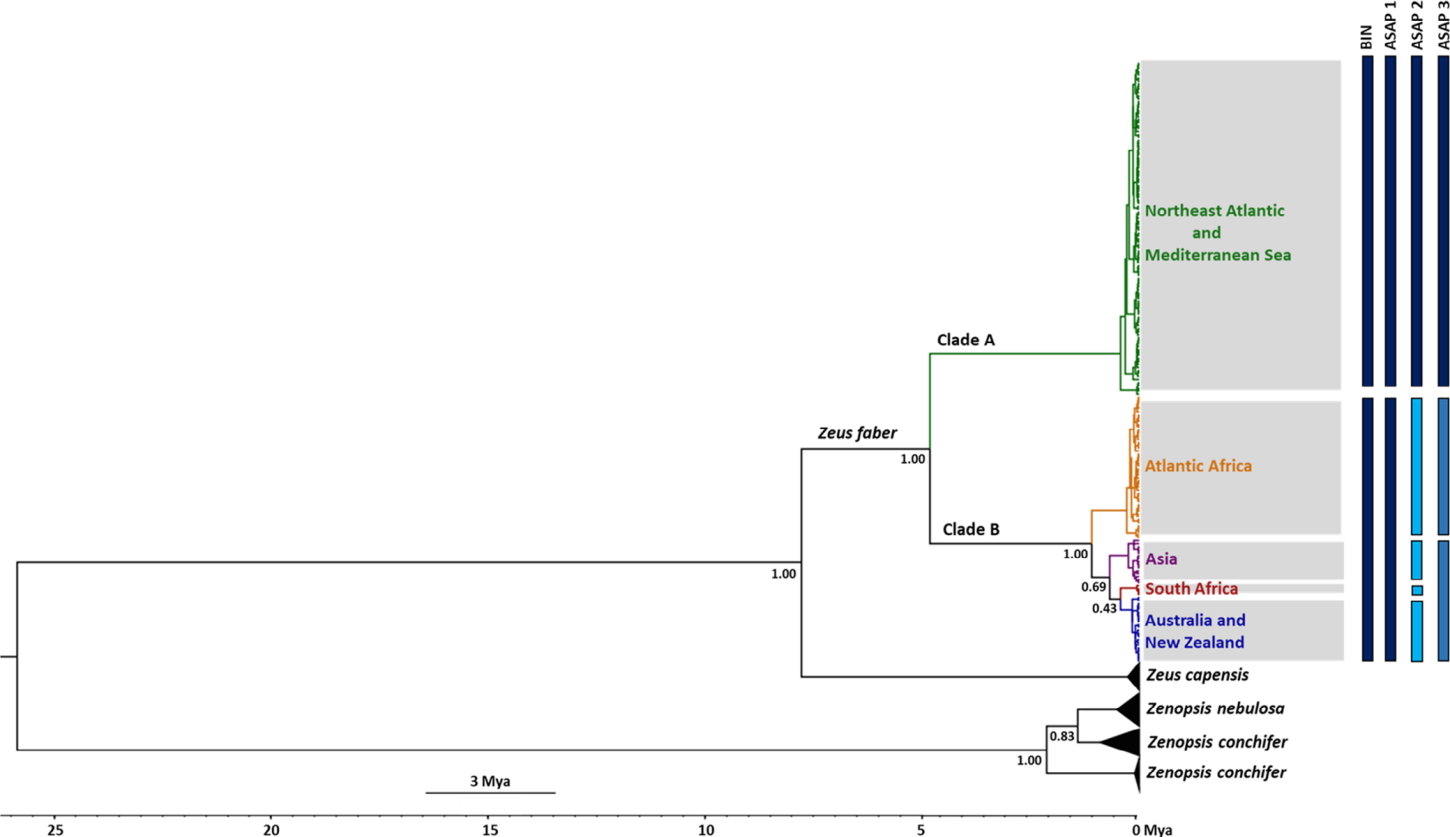
Bayesian tree generated in BEAST for *Zeus faber* based on 620 bp of the COI barcode region. Divergence dates scale are represented in millions of years ago (Mya), whereas node values indicate posterior probabilities. The coloured bars on the right indicate the barcode index number (BIN) partition according to the Refined Single Linkage (RESL) algorithm implemented in the BOLD database and the three Assemble Species by Automatic Partitioning (ASAP) partitions with the lowest ASAP score (2.5).

We analysed a total of 201 *Z. faber* COI sequences, using a 620‐bp alignment with 73 polymorphic sites and 56 PIS (Table 1). Both the reduced‐median haplotype network (Figure 2) and the Bayesian tree (Figure 3) revealed two main highly divergent clades, referred to hereafter as Clade A and Clade B. The two clades displayed a mean K2P distance of 7.4% and a maximum of 8.8% between them. As observed in Table 2, the mean K2P distance was lowest between samples from the Mediterranean and samples from the Northeast Atlantic, with 0.2%. Conversely, the highest mean K2P distances were observed between samples from Asia and the Mediterranean, as well as between Asia and the Northeast Atlantic, both with 7.9%.

**TABLE 1:** Genetic diversity metrics for the entire dataset and the two main clades identified in *Zeus faber* based on 620 bp of the COI barcode region.

	Clade A	Clade B	Overall
*N*	112	89	201
S	17	31	73
PIS	6	20	56
H	18	21	39
Hd	0.641	0.800	0.850
*π*	0.0018	0.0091	0.0365

Abbreviations: H, number of haplotypes; Hd, haplotype diversity; *N*, number of individuals; PIS, number of parsimony‐informative sites; S, number of polymorphic sites; *π*, nucleotide diversity.

**TABLE 2:** Mean K2P distances (% ± standard error) between geographic groups of *Zeus faber* based on 620 bp of the COI barcode region.

Mean K2P (%)	MED	AUS	NEA	ASI	AFR
MED	–				
AUS	7.7 ± 0.006	–			
NEA	0.2 ± 0.004	7.7 ± 0.004	–		
ASI	7.9 ± 0.012	0.9 ± 0.015	7.9 ± 0.007	–	
AFR	7.1 ± 0.004	1.4 ± 0.005	7.1 ± 0.002	1.6 ± 0.008	–
SA	7.4 ± 0.009	1 ± 0.016	7.3 ± 0.006	1.2 ± 0.021	1.7 ± 0.007

*Note*: The regions of sample origin are abbreviated as follows: AFR, Atlantic Africa; ASI, Asia; AUS, Australia and New Zealand; MED, Mediterranean; NEA, Northeast Atlantic; SA, South Africa.

Overall, we found 39 different *Z. faber* haplotypes, with haplotype diversity (Hd) of 0.850 and nucleotide diversity (*π*) of 0.0365 (Table 1). Clade A had a total of 112 sequences and 18 different haplotypes, whereas in Clade B there were 89 sequences and 21 haplotypes. Haplotype diversity (Hd) and nucleotide diversity (*π*) were higher in Clade B compared to Clade A. The genetic diversity metrics calculated for the different geographic regions, as well as the results for Tajima's D test, are represented in Table 3. Haplotype diversity was lowest in the Australia/New Zealand group and highest in the Asia group for both the raw and subsampled data (excluding the South Africa group, which had only one haplotype). In the raw data, nucleotide diversity was lowest for Atlantic Africa and highest for Asia. Conversely, in the subsampled data, nucleotide diversity was lowest for Atlantic Africa but highest for Australia/New Zealand. Statistically significant Tajima's D values (*p* < 0.05) were found solely for Atlantic Africa and Australia/New Zealand in the raw data. In the AMOVA (Table 4), 88.4% of the total genetic variance was attributed to differences between clades A and B, although this was not statistically significant (*p* = 0.067). In contrast, 9.5% of the total variance was attributed to differences among geographic groups within clades, demonstrating highly significant genetic structure (*p* < 0.0001).

**TABLE 3:** Number of samples, genetic diversity metrics and Tajima's D test for the geographic groups of *Zeus faber* based on 620 bp of the COI barcode region.

	*N*	S	PIS	H	Hd	*π*	D	*p*‐Value
Raw data								
Mediterranean	26	7	3	8	0.686	0.0021	−0.879	0.409
Northeast Atlantic	86	13	6	13	0.627	0.0016	−1.686	0.069
Atlantic Africa	48	8	4	10	0.496	0.0010	−1.826	0.046
Australia and New Zealand	16	9	0	4	0.350	0.0018	−2.149	0.008
Asia	12	7	1	5	0.727	0.0023	−1.534	0.114
South Africa	4	*–*	*–*	1	*–*	*–*	*–*	*–*
Subsampled data								
Mediterranean	12	4.6	2.1	5	0.688	0.0021	−0.452	0.633
Northeast Atlantic	12	4.3	1.5	4.4	0.626	0.0016	−0.981	0.369
Atlantic Africa	12	3.2	0.5	4.1	0.501	0.0010	−1.365	0.204
Australia and New Zealand	12	6.8	0	3.2	0.353	0.0028	−1.838	0.06
Asia	12	7	1	5	0.727	0.0023	−1.534	0.114
South Africa	*–*	*–*	*–*	*–*	*–*	*–*	*–*	*–*

Abbreviations: D, Tajima's D; H, number of haplotypes; Hd, haplotype diversity; *N*, number of individuals; PIS, number of parsimony‐informative sites; *p*‐value, Tajima's D *p*‐value; S, number of polymorphic sites; *π*, nucleotide diversity.

**TABLE 4:** The analysis of molecular variance (AMOVA) results for the *Zeus faber* dataset analysed at two hierarchical levels: among clades and among geographic groups within clades (Northeast Atlantic, Mediterranean, Atlantic Africa, South Africa, Australia and New Zealand and Asia).

Source of variation	Df	Sum of squares	Variance components	Percentage of variation (%)	*p*‐Value
Among clades	1	1981.5	20.1	88.4	0.067
Among geographic groups	4	187.4	2.1	9.5	<0.0001
Within geographic groups	186	89.7	0.5	2.1	<0.0001

The maximum K2P distance within Clade A was 0.8%, with a mean of 0.2%. This clade included exclusively sequences from the Northern Hemisphere, namely the Northeast Atlantic Coast of Europe (i.e., Portugal, Spain, United Kingdom, Germany, Denmark, Norway and Sweden), North and Central Atlantic Morocco and the Mediterranean Sea (i.e., France, Greece, Israel, Italy, Malta, Morocco and Turkey). There was no geographic sorting of the haplotypes in Clade A, with the three largest haplogroups including sequences from both aforementioned regions (Figure 2). Maximum K2P within Clade B was 2.3%, with a mean of 0.9%. Clade B comprised sequences from both the Northern and Southern Hemispheres, although with an overall more southern distribution than Clade A. The haplotypes for this clade were, in great part, geographically sorted between four regions (Figure 2). All sequences from Asia (China, Japan, the Philippines and Taiwan) clustered together, along with one sequence lacking geographic information, one from Northwestern Australia and one annotated as originating from the Mid‐Atlantic Bight (a region where *Z. faber*'s distribution is not reported; see Supplementary Text S1 for details). The remaining sequences from Australia and New Zealand formed a cluster that included seven sequences without geographic information. The sequences from South Africa, specifically from the Indian Ocean, formed a group with one haplotype. Sequences from the Atlantic coast of Africa (Angola, Benin, Côte d'Ivoire, Liberia, Guinea‐Bissau, Senegal and Mauritania), including only the South Atlantic region of Morocco, clustered together. The transition zone between the two clades was found along the Atlantic coast of Morocco, approximately extending from south of Cape Ghir (30.63° N, 9.89° W) to Cape Boujdour (26.13° N, 14.50° W) (Figure 1).

Species delimitation analyses supported the existence of distinct genetic groupings (Figure 3). In the BOLD database, Clade A corresponded to BIN BOLD:AGC9721, whereas Clade B corresponded to BOLD:AAA7904 (BOLD accessed 15 January 2025). The ASAP analysis produced three optimal partitions based on the lowest ASAP score (2.5): Partition 1 identified two MOTUs, corresponding to clades A and B; partition 2 identified five MOTUs, corresponding to Clade A plus the four lineages within Clade B (Atlantic Africa, South Africa, Australia/New Zealand and Asia); and partition 3 identified three MOTUs, representing Clade A, the Atlantic Africa lineage within Clade B and a subclade encompassing the Asia, Australia/New Zealand and South Africa lineages.

**FIGURE 4:**
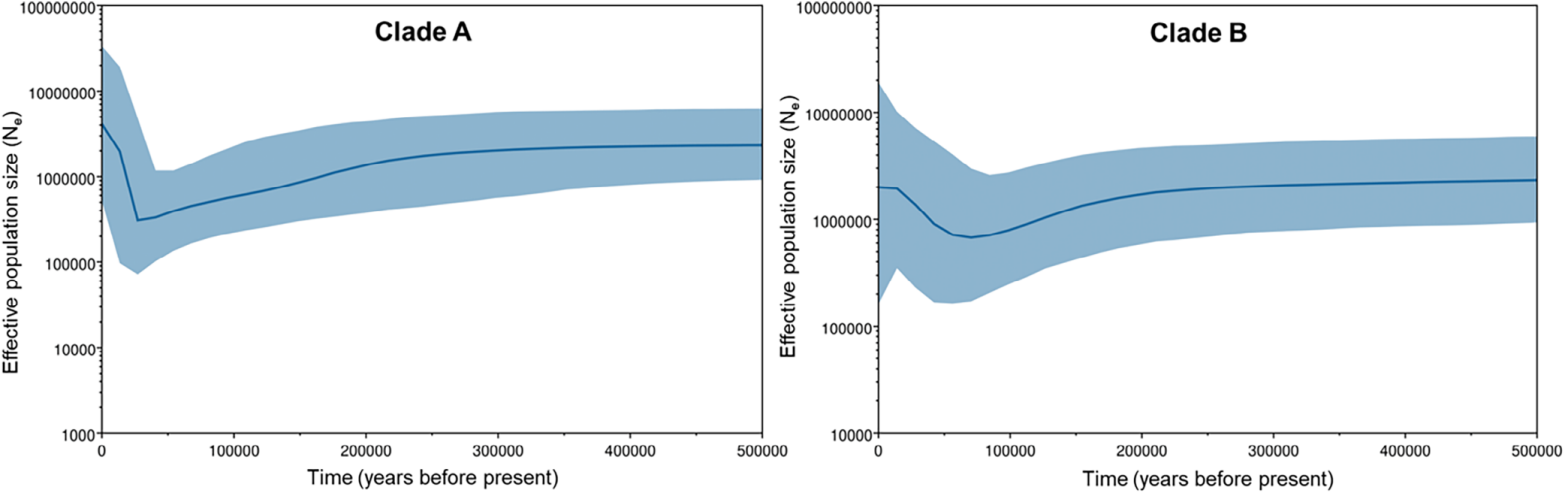
Bayesian Skyline Plots (BSPs) for Clade A and Clade B of *Zeus faber* based on 620 bp of the COI barcode region. The *x*‐axis represents years before present, and the *y*‐axis represents effective population size (*N*_*e*_).

BSPs were used to estimate changes in effective population size over time for clades A and B based on coalescent data derived from DNA barcodes (Figure 4). The analysis focused on the last 500,000 years, as no coalescent points were observed beyond this period. For Clade A, the BSP revealed a sharp expansion in effective population size, beginning approximately 20,000 years ago, whereas Clade B showed a more gradual increase starting around 70,000 years ago.

## 4 DISCUSSION

This phylogeographic study provided novel insights into the evolutionary history and genetic structure of *Z. faber*, a widely distributed and commercially important marine fish species. To conduct the analyses, we employed the COI DNA barcode region, a well‐established marker with recognized merits for fish identification (Arroyave et al., 2019; Bingpeng et al., 2018; Guimarães et al., 2022; Landi et al., 2014; Shakouri et al., 2023). The vast public availability of DNA barcodes from specimens collected worldwide (Porter & Hajibabaei, 2018; Ratnasingham et al., 2024) allowed us to conduct this study on a geographically wide scale, which would have been logistically more challenging using other genetic markers. Our investigation was prompted by *Z. faber*'s previously observed high intraspecific genetic divergence, along with its wide geographic distribution and large DNA barcode data gap along the Atlantic coast of Africa.

The phylogeographic reconstruction revealed the presence of two deeply divergent clades – Clade A and Clade B – with a mean K2P of 7.4%. This level of genetic divergence largely surpassed the levels of genetic variation generally observed in marine fish species (Costa et al., 2012; Dettai et al., 2011; Knebelsberger et al., 2014; Ward et al., 2005). Our results confirmed and expanded on the previous screening for genetic divergence of *Z. faber* by Ward et al. (2008), incorporating sequences from additional geographic regions and a robust phylogeographic analysis. These findings clearly suggested that specimens identified morphologically as *Z. faber* belong to at least two sister taxa, with the widely distributed Clade B exhibiting a clear phylogeographic pattern. Moreover, by expanding DNA barcode sampling along Africa's Atlantic shores, we clarified the mitochondrial clade of these specimens and mapped the transition zone along Morocco's Atlantic coast. This has improved our understanding of the distribution of both clades in the Eastern Atlantic Ocean.

Regarding *Z. faber*'s evolutionary history, according to the reduced‐median network and the Bayesian analysis (Figures 2 and 3), the two clades likely diverged from a common ancestor in the Eastern Atlantic Ocean. Clade A lacked geographic structure and exhibited a high degree of haplotypes shared between specimens from the Northeast Atlantic Ocean and the Mediterranean Sea, along with a comparatively lower mean K2P distance between the two regions (0.2%, Table 2). These patterns suggested some degree of genetic connectivity, potentially indicating gene flow between the two regions. On the contrary, Clade B exhibited a considerable degree of phylogeographic structure with four distinct lineages identified (Figures 2 and 3). Moreover, Clade B showed moderate divergence between geographic regions (Table 2), with the maximum K2P distance having been observed between a sequence from China and a sequence from Angola (2.3%). Comparatively, the Bayesian analysis indicated that Clade B began diverging at an earlier stage than Clade A, which was consistent with its more defined geographic structure. This structure was further supported by the AMOVA (Table 4), which revealed that 9.5% of the total genetic variance was attributed to differences among the previously defined geographic groups. Although this percentage was relatively low compared to the variance observed between clades, it was statistically significant, demonstrating consistent and detectable geographic differentiation. The variance between clades A and B was non‐significant (*p* = 0.067) likely due to the limited degrees of freedom (df = 1). However, it still accounted for 88.4% of the total genetic variance, suggesting strong genetic differentiation despite not reaching statistical significance.

The reduced‐median network and Bayesian tree presented differing perspectives on the relationships within Clade B. In the network, Australia/New Zealand and Asia appeared more closely related, whereas the Bayesian tree showed Australia/New Zealand grouping with South Africa. These differences could have been attributed to the distinct analytical approaches used. The reduced‐median network relied on parsimony, favouring the simplest explanation with the fewest evolutionary changes, whereas the Bayesian tree employed a probabilistic framework. The lower posterior probabilities for the Clade B branches in the tree further indicated uncertainty in these specific evolutionary relationships, suggesting that they were unresolved with the current dataset. Indeed, one of the MOTU partitions found by ASAP (Figure 3) clustered the three lineages from Australia/New Zealand, South Africa and Asia into a single MOTU, reflecting this uncertainty. Thus, future research incorporating additional genetic markers could improve resolution and clarify the evolutionary history of this taxon, similar to efforts in the *M. cephalus* species complex (Thieme et al., 2025).

Although the divide between the Northeast Atlantic Ocean and the Mediterranean Sea has been reported as a phylogeographic break for fish species (Bargelloni et al., 2003; Larmuseau et al., 2009; McKeown et al., 2020; Patarnello et al., 2007), this pattern did not occur for *Z. faber*. Similarly, Cunha et al. (2022) found haplotype sharing between samples from the Atlantic Ocean and the Mediterranean for the bogue *Boops boops* (L. 1758) while reporting genetic structure within the Eastern Atlantic. This parallel suggested that, like *B. boops*, *Z. faber* did not exhibit a phylogeographic break between these two regions. Alternatively, the transition zone between the two *Z. faber* clades occurred within the Atlantic coast of Morocco, approximately extending from south of Cape Ghir to Cape Boujdour (Figure 1). This phylogeographic break has already been noticed for species such as *Sardina pilchardus* (Walbaum 1792), *M. cephalus* or close to this area for *Engraulis encrasicolus* (L. 1758) (Chahdi Ouazzani et al., 2017; Chlaida et al., 2009; Thieme et al., 2025).

Based on our sampling, Clade A and Clade B appeared to exist in allopatry, with no evidence of overlap between their distributions. Moreover, we were unable to find records of *Z. faber* from the transition zone along the Atlantic coast of Morocco. Yet, it was unclear whether this reflected a true absence or insufficient exploration of the area, particularly in deeper habitats beyond the usual survey depths. This sharp geographic isolation between the clades was unusual for marine fishes, and the complex upwelling systems along the Moroccan Atlantic coast may have contributed to this separation. Seasonal upwelling around Cape Ghir and more persistent upwelling further South, around Cape Juby (Makaoui et al., 2012, 2015), likely created dynamic environmental gradients that maintained the separation of the two clades. By altering water temperature and nutrient availability (Barton et al., 1998), these upwelling systems can influence marine habitat structure (Largier, 2020; Tapia et al., 2009). This may have limited the geographic overlap between the two clades and contributed to their genetic separation. In addition, the increased biological productivity associated with these upwelling systems (Moore et al., 2004; Radenac et al., 2020) may have further enhanced habitat variability in the region. This, in turn, could have contributed to shaping the distribution patterns and genetic differentiation observed between the two *Z. faber* clades. Similar oceanographic processes have been shown to sustain genetic structure across large geographic areas. For instance, in the *M. cephalus* species complex in the Northwest Pacific, significant genetic differentiation has been documented between cryptic species likely influenced by strong current systems, temperature gradients and different reproductive behaviour that have maintained the genetic integrity of these species despite secondary contact after Pleistocene vicariant events (Shen et al., 2011, 2015).

Although the upwelling systems likely contributed to maintaining or reinforcing the geographic separation between Clade A and Clade B, it was uncertain whether these oceanographic features were present during their initial divergence from a common ancestor. This suggested that other historical factors may have influenced this divergence. One hypothesis is the potential role of the Messinian salinity crisis (MSC), which took place between 5.96 and 5.33 million years ago (Briand, 2008; Krijgsman et al., 1999). The coalescence time for the two *Z. faber* clades, estimated at 4.81 million years ago (95% CI: 3.89–5.84 million years), coincided with the aftermath of this event. Although the MSC primarily affected the Mediterranean basin, it also disrupted the hydrological balance in adjacent Atlantic waters (Blanc & Duplessy, 1982; Mascle & Mascle, 2019). These environmental changes, particularly after the reopening of the Gibraltar Strait, would have reshaped marine habitats and contributed to the genetic isolation of marine populations. The MSC could have contributed to the divergence of Clade A and Clade B from a common ancestor in the tropical‐subtropical Northeast Atlantic. However, this hypothesis requires further investigation to assess its validity. To evaluate the plausibility of this hypothesis, future studies could use additional genetic markers to refine divergence time estimates, model paleoclimate and oceanographic changes during the MSC, examine sediment and fossil records for evidence of habitat shifts and compare biogeographic patterns across taxa in the region.

The BSP (Figure 4) for Clade A indicated a rapid population expansion starting approximately 20 thousand years ago, a pattern that coincided with the environmental changes of the Last Glacial Maximum (LGM), occurring between 19 and 26.5 thousand years ago (Clark et al., 2009). The significant shifts in sea levels and rising ocean temperatures during this period likely expanded habitat availability and created more favourable environmental conditions. This would have allowed populations to colonize previously inaccessible or unfavourable areas, particularly in the Northeast Atlantic and Mediterranean regions. This pattern, along with considerable haplotype sharing and a lack of clear geographic structure in Clade A, suggested that the expansion could have originated from a smaller founding population that was constrained by prior conditions. In contrast, the BSP for Clade B indicated a more gradual population growth beginning around 70 thousand years ago, possibly shaped by more stable environmental conditions and subtle ecological pressures across its broader and more southern geographic range. Although statistical significance was found only in the raw data, negative Tajima's *D* values were observed in Clade B for regions such as Australia/New Zealand and Atlantic Africa, consistent with signals of population expansion within these areas. Together, these patterns further suggested distinct demographic histories for clades A and B shaped by differing environmental conditions and pressures.

Genetic diversity metrics revealed some regional differences (Table 3). Due to limited sampling, no definitive conclusions could be drawn about genetic diversity in South Africa, where only four samples were analysed, represented by a single haplotype. Australia and New Zealand exhibited the lowest haplotype count and haplotype diversity in both the raw and subsampled data. Despite comparable nucleotide diversity, the reduced haplotype diversity in these regions suggested that, although the few haplotypes exhibited some genetic variation, the population's overall genetic diversity was reduced. Similarly, Atlantic Africa was comparatively low in terms of genetic diversity metrics (both raw and subsampled) despite the considerable number of samples collected from different sites along the West African coast. Reduced genetic diversity can limit adaptability to environmental changes, necessitating targeted management strategies for certain regions (Domingues et al., 2018; Ferrette et al., 2021; Frankham, 2005). Future studies should focus on expanding sampling efforts across underrepresented regions to enhance resolution and enable a more comprehensive assessment of population stability and genetic diversity.

## 5 CONCLUSIONS

Our study reinforced the presence of two highly divergent clades for the *Z. faber* morphospecies, with distinct geographic distributions and evolutionary histories. Moreover, it showed that Clade B consisted of at least four lineages with noteworthy phylogeographic structure. By mapping the transition zone between the two clades along the Atlantic coast of Morocco and considerably increasing the sampling across several African countries on the Eastern Atlantic, we have provided a clearer understanding of their distribution and evolutionary history. Although DNA barcodes suggested the presence of at least two species under *Z. faber*, further studies using morphological analysis, nuclear markers and techniques like restriction site‐associated DNA markers (Jones et al., 2013; Labrador et al., 2022) are needed to confirm its taxonomic status and clarify evolutionary relationships. Even though *Z. faber* is a commercially important species, its distinctive morphology – an unusual body shape, very long dorsal‐fin rays and an iconic large dark spot surrounded by a light ring on each side – may have occasionally facilitated its identification worldwide through superficial visual inspections. Thus, considering the genetic differentiation reported in this study, synonymized and invalid *Zeus* species’ names likely deserve a closer morphological re‐inspection. Moreover, expanding the sampling and generating DNA barcodes from additional regions will help refine the delimitation of the two clades and may uncover additional ones. Independently of the conclusions on the species status, the genetic structure here reported has potential implications for fisheries management and conservation that must be considered. Particularly in Morocco, recognizing the existence of these two clades in stock assessment is crucial, as overlooking their genetic distinction could lead to inaccurate evaluations of population health and overestimation of stock sizes (Hutchings & Reynolds, 2004; Ruzzante et al., 1997). Fisheries management in other regions should also consider these findings, as the clades and lineages here reported may have relevance for stock delimitation, fisheries sustainability, global fisheries statistics and seafood authentication.

## AUTHOR CONTRIBUTIONS

Conceptualization: João Tadeu Fontes, Kenza Mokhtar‐Jamaï, Jean‐Dominique Durand, Pedro Soares and Filipe Oliveira Costa; methodology: João Tadeu Fontes, Kenza Mokhtar‐Jamaï, Jean‐Dominique Durand, Pedro Soares and Filipe Oliveira Costa; formal analysis: João Tadeu Fontes and Pedro Soares; data acquisition and processing: João Tadeu Fontes, Kenza Mokhtar‐Jamaï, Zakariya Nchioua, Jean‐Dominique Durand, Monica Landi, João Meira, Luís Machado, Ayoub Baali, Ignacio Sobrino, Emmanuel Kouamé, Béatrice Abouo Adepo‐Gourène, Mamadou Diop, Néné Gallé Kidé, Austin Saye Wehye and Zacharie Sohou; resources: Kenza Mokhtar‐Jamaï, Jean‐Dominique Durand, Iça Barri, Ignacio Sobrino, Miguel Carneiro, Rogélia Martins and Filipe Oliveira Costa; writing – original draft: João Tadeu Fontes; writing – review and editing: João Tadeu Fontes, Kenza Mokhtar‐Jamaï, Jean‐Dominique Durand, João Meira, Luís Machado, Ayoub Baali, Emmanuel Kouamé, Zacharie Sohou, Pedro Soares and Filipe Oliveira Costa. All authors reviewed and approved the final version of the manuscript.

## FUNDING INFORMATION

This work was funded by the project ‘FISH‐DNA‐MONITOR: Supporting monitoring and management of fisheries resources in Guinea‐Bissau through capacity‐building on cutting‐edge DNA‐based approaches’ (FCT AGA‐KHAN/541703280/2019) funded by the Portuguese Foundation of Science and Technology (FCT, I.P.) and Aga Khan Development Network, by the CBMA ‘Contrato‐Programa’ (UIDB/04050/2020; https://doi.org/10.54499/UIDB/04050/2020), and by the ARNET ‘Contrato‐Programa’ (LA/P/0069/2020; https://doi.org/10.54499/LA/P/0069/2020), funded by national funds through FCT I.P. J.T.F. (UI/BD/150910/2021; https://doi.org/10.54499/UI/BD/150910/2021) was supported by the Collaboration Protocol for Financing the Multiannual Research Grants Plan for Doctoral Students with financial support from FCT I.P. and the European Social Fund under the Northern Regional Operational Program – Norte2020. The generation of sequence data from specimens collected in Senegal, Mauritania and Côte d'Ivoire was funded by IRD through the WAMBA‐Net International Research Network.

## Supporting information


**Data S1.** Supporting information.

## References

[jfb70245-bib-0001] Arroyave, J. , Martinez, C. M. , & Stiassny, M. L. (2019). DNA barcoding uncovers extensive cryptic diversity in the African long‐fin tetra Bryconalestes longipinnis (Alestidae: Characiformes). Journal of Fish Biology, 95, 379–392. 31001832 10.1111/jfb.13987

[jfb70245-bib-0002] Avise, J. C. , Bowen, B. W. , & Ayala, F. J. (2016). In the light of evolution X: Comparative phylogeography. Proceedings of the National Academy of Sciences of the United States of America, 113, 7957–7961. 27432955 10.1073/pnas.1604338113 PMC4961136

[jfb70245-bib-0003] Avise, J. C. , Brick, K. , & Fisher, M. (2000). Phylogeography: The history and formation of species. Harvard University Press.

[jfb70245-bib-0004] Bandelt, H.‐J. , Forster, P. , Sykes, B. C. , & Richards, M. B. (1995). Mitochondrial portraits of human populations using median networks. Genetics, 141, 743–753. 8647407 10.1093/genetics/141.2.743 PMC1206770

[jfb70245-bib-0005] Bargelloni, L. , Alarcon, J. , Alvarez, M. , Penzo, E. , Magoulas, A. , Reis, C. , & Patarnello, T. (2003). Discord in the family Sparidae (Teleostei): Divergent phylogeographical patterns across the Atlantic–Mediterranean divide. Journal of Evolutionary Biology, 16, 1149–1158. 14640406 10.1046/j.1420-9101.2003.00620.x

[jfb70245-bib-0006] Barton, E. D. , Arístegui, J. , Tett, P. , Cantón, M. , García‐Braun, J. , Hernández‐León, S. , Nykjaer, L. , Almeida, C. , Almunia, J. , Ballesteros, S. , Basterretxea, G. , Escánez, J. , García‐Weill, L. , Hernández‐Guerra, A. , López‐Laatzen, F. , Molina, R. , Montero, M. F. , Navarro‐Pérez, E. , Rodríguez, J. M. , … Wild, K. (1998). The transition zone of the canary current upwelling region. Progress in Oceanography, 41, 455–504.

[jfb70245-bib-0007] Becker, R. A. , Wilks, A. R. , & Brownrigg, R. (2022). maps: Draw Geographical Maps, 2022.

[jfb70245-bib-0008] Bermingham, E. , McCafferty, S. S. , & Martin, A. P. (1997). Fish biogeography and molecular clocks: Perspectives from the Panamanian isthmus. In Molecular systematics of fishes (pp. 113–128). Academic Press.

[jfb70245-bib-0009] Bingpeng, X. , Heshan, L. , Zhilan, Z. , Chunguang, W. , Yanguo, W. , & Jianjun, W. (2018). DNA barcoding for identification of fish species in the Taiwan Strait. PLoS One, 13, e0198109. 29856794 10.1371/journal.pone.0198109 PMC5983523

[jfb70245-bib-0010] Blanc, P.‐L. , & Duplessy, J.‐C. (1982). The deep‐water circulation during the Neogene and the impact of the Messinian salinity crisis. Deep‐Sea Research Part A: Oceanographic Research Papers, 29, 1391–1414.

[jfb70245-bib-0011] Briand, F. (2008). The Messinian salinity crisis from mega‐deposits to microbiology—A consensus report (Overview). In CIESM Workshop Monographs (Vol. 33, pp. 7–28). CIESM (Commission Internationale pour l’Exploration Scientifique de la Mer Méditerranée).

[jfb70245-bib-0012] Caballero‐Huertas, M. , Frigola‐Tepe, X. , Coll, M. , Muñoz, M. , & Viñas, J. (2022). The current knowledge status of the genetic population structure of the European sardine (Sardina pilchardus): Uncertainties to be solved for an appropriate fishery management. Reviews in Fish Biology and Fisheries, 32, 745–763.

[jfb70245-bib-0013] Cabezas, M. P. , Lasso‐Alcalá, O. M. , Quintero‐T, E. , Xavier, R. , Giarrizzo, T. , Nunes, J. L. S. , Machado, F. S. , Gómez, J. , Silva Pedroza, W. , & Jowers, M. J. (2022). Clarifying the taxonomy of some cryptic blennies (Blenniidae) in their native and introduced range. Scientific Reports, 12, 9514. 35680914 10.1038/s41598-022-12580-z PMC9184548

[jfb70245-bib-0014] Catarino, D. , Stefanni, S. , Jorde, P. E. , Menezes, G. M. , Company, J. B. , Neat, F. , & Knutsen, H. (2017). The role of the strait of Gibraltar in shaping the genetic structure of the Mediterranean grenadier, Coryphaenoides mediterraneus, between the Atlantic and Mediterranean Sea. PLoS One, 12, e0174988. 28459855 10.1371/journal.pone.0174988 PMC5411037

[jfb70245-bib-0015] Chahdi Ouazzani, K. , Benazzou, T. , Charouki, N. , Bonhomme, F. , & Chlaida, M. (2017). Genetic differentiation of European anchovy (Engraulis encrasicolus) along the Moroccan coast reveals a phylogeographic break around the 25th parallel north. Marine Biology Research, 13, 342–350.

[jfb70245-bib-0016] Chang, C.‐H. , Shao, K.‐T. , Lin, H.‐Y. , Chiu, Y.‐C. , Lee, M.‐Y. , Liu, S.‐H. , & Lin, P.‐L. (2017). DNA barcodes of the native ray‐finned fishes in Taiwan. Molecular Ecology Resources, 17, 796–805. 27717215 10.1111/1755-0998.12601

[jfb70245-bib-0017] Chlaida, M. , Laurent, V. , Kifani, S. , Benazzou, T. , Jaziri, H. , & Planes, S. (2009). Evidence of a genetic cline for Sardina pilchardus along the northwest African coast. ICES Journal of Marine Science, 66, 264–271.

[jfb70245-bib-0018] Clark, P. U. , Dyke, A. S. , Shakun, J. D. , Carlson, A. E. , Clark, J. , Wohlfarth, B. , Mitrovica, J. X. , Hostetler, S. W. , & McCabe, A. M. (2009). The Last glacial maximum. Science, 2009(325), 710–714. 10.1126/science.1172873 19661421

[jfb70245-bib-0019] Costa, F. O. , Landi, M. , Martins, R. , Costa, M. H. , Costa, M. E. , Carneiro, M. , Alves, M. J. , Steinke, D. , & Carvalho, G. R. (2012). A ranking system for reference libraries of DNA barcodes: Application to marine fish species from Portugal. PLoS One, 7, e35858. 22558244 10.1371/journal.pone.0035858 PMC3338485

[jfb70245-bib-0020] Crosetti, D. , Avise, J. C. , Placidi, F. , Rossi, A. R. , & Sola, L. (1993). Geographic variability in the grey mullet Mugil cephalus: Preliminary results of mtDNA and chromosome analyses. In Genetics in aquaculture IV (Vol. 111, pp. 95–101). Elsevier.

[jfb70245-bib-0021] Cunha, R. L. , Faleh, A. B. , Francisco, S. , Šanda, R. , Vukić, J. , Corona, L. , & Castilho, R. (2022). Three mitochondrial lineages and no Atlantic‐Mediterranean barrier for the bogue Boops boops across its widespread distribution. Scientific Reports, 12, 22124. 36543927 10.1038/s41598-022-26651-8 PMC9772343

[jfb70245-bib-0022] Dalongeville, A. , Nielsen, E. S. , Teske, P. R. , & von Der Heyden, S. (2022). Comparative phylogeography in a marine biodiversity hotspot provides novel insights into evolutionary processes across the Atlantic‐Indian Ocean transition. Diversity and Distributions, 28, 2622–2636.

[jfb70245-bib-0023] Darriba, D. , Taboada, G. L. , Doallo, R. , & Posada, D. (2012). jModelTest 2: More models, new heuristics and parallel computing. Nature Methods, 9, 772. 10.1038/nmeth.2109 PMC4594756 22847109

[jfb70245-bib-0024] Dettai, A. , Lautredou, A.‐C. , Bonillo, C. , Goimbault, E. , Busson, F. , Causse, R. , Couloux, A. , Cruaud, C. , Duhamel, G. , Hautecoeur, M. , Iglesias, S. , Koubbi, P. , Lecointre, G. , Moteki, M. , Pruvost, P. , Tercerie, S. , Ozouf, C. , & Denys, G. (2011). The actinopterygian diversity of the CEAMARC cruises: Barcoding and molecular taxonomy as a multi‐level tool for new findings. Deep Sea Research Part II: Topical Studies in Oceanography, 58, 250–263.

[jfb70245-bib-0025] Domingues, R. R. , Hilsdorf, A. W. S. , & Gadig, O. B. F. (2018). The importance of considering genetic diversity in shark and ray conservation policies. Conservation Genetics, 19, 501–525.

[jfb70245-bib-0026] Dunn, M. (2001). The biology and exploitation of John dory, *Zeus faber* (Linnaeus, 1758) in the waters of England and Wales. ICES Journal of Marine Science, 58, 96–105.

[jfb70245-bib-0027] Excoffier, L. , Laval, G. , & Schneider, S. (2007). Arlequin (version 3.0): An integrated software package for population genetics data analysis. Evolutionary Bioinformatics Online, 1, 47–50. 19325852 PMC2658868

[jfb70245-bib-0028] Excoffier, L. , Smouse, P. E. , & Quattro, J. M. (1992). Analysis of molecular variance inferred from metric distances among DNA haplotypes: Application to human mitochondrial DNA restriction data. Genetics, 131, 479–491. 1644282 10.1093/genetics/131.2.479 PMC1205020

[jfb70245-bib-0029] Ferrette, B. L. d. S. , Mourato, B. , Hazin, F. H. V. , Arocha, F. , Williams, S. M. , Rodrigues Junior, C. E. , Porto‐Foresti, F. , de Amorim, A. F. , Rotundo, M. M. , Coelho, R. , Hoolihan, J. P. , Sow, F. N. , Diaha, N.’g. C. , Romanov, E. V. , Domingues, R. R. , Oliveira, C. , Foresti, F. , & Mendonça, F. F. (2021). Global phylogeography of sailfish: Deep evolutionary lineages with implications for fisheries management. Hydrobiologia, 848(17), 3883–3904.

[jfb70245-bib-0030] Frankham, R. (2005). Genetics and extinction. Biological Conservation, 126, 131–140.

[jfb70245-bib-0031] Gascuel, D. , Labrosse, P. , Meissa, B. , Taleb Sidi, M. O. , & Guénette, S. (2007). Decline of demersal resources in north‐West Africa: An analysis of Mauritanian trawl‐survey data over the past 25 years. African Journal of Marine Science, 29, 331–345.

[jfb70245-bib-0032] Giarratana, F. , Ziino, G. , D'Andrea, V. , Panebianco, A. , & Giuffrida, A. (2018). Quality assessment of Zeus faber (Peter's fish) ovaries regularly commercialized for human consumption. Italian Journal of Food Safety, 7(1), 6997. 29732333 10.4081/ijfs.2018.6997 PMC5913707

[jfb70245-bib-0033] Grant, W. , & Bowen, B. W. (1998). Shallow population histories in deep evolutionary lineages of marine fishes: Insights from sardines and anchovies and lessons for conservation. Journal of Heredity, 89, 415–426.

[jfb70245-bib-0034] Guimarães, K. L. A. , Lima, M. P. , Santana, D. J. , de Souza, M. F. B. , Barbosa, R. S. , & Rodrigues, L. R. R. (2022). DNA barcoding and phylogeography of the Hoplias malabaricus species complex. Scientific Reports, 12, 1–15. 35347184 10.1038/s41598-022-09121-z PMC8960906

[jfb70245-bib-0035] Hüne, M. , Oyarzún, P. A. , Reyes, P. , Rivera, G. , & Montecinos, M. (2021). Phylogeographic analysis of Thyrsites atun (Perciformes: Gempylidae) reveals connectivity between fish from South Africa and Chile. Marine Biology Research, 17, 401–413.

[jfb70245-bib-0036] Hutchings, J. A. , & Reynolds, J. D. (2004). Marine fish population collapses: Consequences for recovery and extinction risk. Bioscience, 54, 297–309.

[jfb70245-bib-0037] Hyde, J. R. , Underkoffler, K. E. , & Sundberg, M. A. (2014). DNA barcoding provides support for a cryptic species complex within the globally distributed and fishery important opah (Lampris guttatus). Molecular Ecology Resources, 14, 1239–1247. 24751335 10.1111/1755-0998.12268

[jfb70245-bib-0038] Ismen, A. , Arslan, M. , Yigin, C. C. , & Bozbay, N. A. (2013). Age, growth, reproduction and feeding of John dory, Z eus faber (Pisces: Zeidae), in the Saros Bay (North Aegean Sea). Journal of Applied Ichthyology, 29, 125–131.

[jfb70245-bib-0039] Ivanova, N. V. , Zemlak, T. S. , Hanner, R. H. , & Hebert, P. D. (2007). Universal primer cocktails for fish DNA barcoding. Molecular Ecology Notes, 7, 544–548.

[jfb70245-bib-0040] Iwamoto, T. (2015). Zeus faber. The IUCN Red List of Threatened Species, 2015, e.T198769A42390771.

[jfb70245-bib-0041] Jamaludin, N.‐A. , Mohd‐Arshaad, W. , Mohd Akib, N. A. , Zainal Abidin, D.‐H. , Nghia, N. V. , & Nor, S.‐A. M. (2020). Phylogeography of the Japanese scad, Decapterus maruadsi (Teleostei; Carangidae) across the central Indo‐West Pacific: Evidence of strong regional structure and cryptic diversity. Mitochondrial DNA Part A DNA Mapping, Sequencing, and Analysis, 31, 298–310. 32744461 10.1080/24701394.2020.1799996

[jfb70245-bib-0042] Janssen, G. (1979). The occurrence of Zeus faber (Linnaeus, 1758) in the coastal waters of The Netherlands (Pisces, Zeiformes). Bulletin Zoölogisch Museum, 6, 153–155.

[jfb70245-bib-0043] Jones, B. A. , Grace, D. , Kock, R. , Alonso, S. , Rushton, J. , Said, M. Y. , McKeever, D. , Mutua, F. , Young, J. , McDermott, J. , & Pfeiffer, D. U. (2013). Zoonosis emergence linked to agricultural intensification and environmental change. Proceedings of the National Academy of Sciences, 110, 8399–8404. 10.1073/pnas.1208059110 PMC3666729 23671097

[jfb70245-bib-0044] Kim, H. J. , Kim, H.‐G. , & Oh, C.‐W. (2020). Diet composition and feeding strategy of John dory, Zeus faber, in the coastal waters of Korea. Journal of Ecology and Environment, 44, 1–8.

[jfb70245-bib-0045] Kimura, M. (1980). A simple method for estimating evolutionary rates of base substitutions through comparative studies of nucleotide sequences. Journal of Molecular Evolution, 16, 111–120. 7463489 10.1007/BF01731581

[jfb70245-bib-0046] Knebelsberger, T. , Landi, M. , Neumann, H. , Kloppmann, M. , Sell, A. F. , Campbell, P. D. , & Costa, F. O. (2014). A reliable DNA barcode reference library for the identification of the north European shelf fish fauna. Molecular Ecology Resources, 14, 1060–1071. 24618145 10.1111/1755-0998.12238

[jfb70245-bib-0047] Kottillil, S. , Rao, C. , Bowen, B. W. , & Shanker, K. (2023). Phylogeography of sharks and rays: A global review based on life history traits and biogeographic partitions. PeerJ, 11, e15396. 37283899 10.7717/peerj.15396 PMC10239618

[jfb70245-bib-0048] Krijgsman, W. , Hilgen, F. J. , Raffi, I. , Sierro, F. J. , & Wilson, D. (1999). Chronology, causes and progression of the Messinian salinity crisis. Nature, 400, 652–655.

[jfb70245-bib-0049] Labrador, K. , Palermo, J. , Agmata, A. , Ravago‐Gotanco, R. , & Pante, M. J. (2022). Restriction site‐associated DNA sequencing reveals local adaptation despite high levels of gene flow in Sardinella lemuru (Bleeker, 1853) along the northern coast of Mindanao, Philippines. Frontiers in Marine Science, 9, 766936.

[jfb70245-bib-0050] Landi, M. , Dimech, M. , Arculeo, M. , Biondo, G. , Martins, R. , Carneiro, M. , Carvalho, G. R. , Lo Brutto, S. , & Costa, F. O. (2014). DNA barcoding for species assignment: The case of Mediterranean marine fishes. PLoS One, 9, e106135. 25222272 10.1371/journal.pone.0106135 PMC4164363

[jfb70245-bib-0051] Largier, J. L. (2020). Upwelling bays: How coastal upwelling controls circulation, habitat, and productivity in bays. Annual Review of Marine Science, 12, 415–447. 10.1146/annurev-marine-010419-011020 31530079

[jfb70245-bib-0052] Larmuseau, M. H. , Van Houdt, J. K. , Guelinckx, J. , Hellemans, B. , & Volckaert, F. A. (2009). Distributional and demographic consequences of Pleistocene climate fluctuations for a marine demersal fish in the north‐eastern Atlantic. Journal of Biogeography, 36, 1138–1151.

[jfb70245-bib-0053] Liu, B. , Yang, J.‐W. , Liu, B.‐S. , Zhang, N. , Guo, L. , Guo, H.‐Y. , & Zhang, D.‐C. (2022). Detection and identification of marine fish mislabeling in Guangzhou's supermarkets and sushi restaurants using DNA barcoding. Journal of Food Science, 87, 2440–2449. 35438192 10.1111/1750-3841.16150

[jfb70245-bib-0054] Makaoui, A. , Idrissi, M. , Benazzouz, A. , Laamel, M. A. , Agouzouk, A. , Larissi, J. , & Hilmi, K. (2015). Hydrological variability of the upwelling and the filament in cape Juby (28 N) Morocco. International Journal, 3, 9–26.

[jfb70245-bib-0055] Makaoui, A. , Orbi, A. , Arestigui, J. , Azzouz, A. B. , Laarissi, J. , Agouzouk, A. , & Hilmi, K. (2012). Hydrological seasonality of cape Ghir filament in Morocco. Natural Science, 4, 5–13.

[jfb70245-bib-0056] Martins, N. T. , Macagnan, L. B. , Cassano, V. , & Gurgel, C. F. D. (2022). Brazilian marine phylogeography: A literature synthesis and analysis of barriers. Molecular Ecology, 31, 5423–5439. 36073087 10.1111/mec.16684

[jfb70245-bib-0057] Mascle, G. , & Mascle, J. (2019). The Messinian salinity legacy: 50 years later. Mediterranean Geoscience Reviews, 1, 5–15.

[jfb70245-bib-0058] McKeown, N. J. , Gwilliam, M. P. , Healey, A. J. , Skujina, I. , Potts, W. M. , Sauer, W. H. , & Shaw, P. W. (2020). Deep phylogeographic structure may indicate cryptic species within the Sparid genus Spondyliosoma. Journal of Fish Biology, 96, 1434–1443. 32154919 10.1111/jfb.14316

[jfb70245-bib-0059] Moore, J. K. , Doney, S. C. , & Lindsay, K. (2004). Upper ocean ecosystem dynamics and iron cycling in a global three‐dimensional model. Global Biogeochemical Cycles, 18, 2004GB002220.

[jfb70245-bib-0060] Nneji, L. M. , Adeola, A. C. , Mustapha, M. K. , Oladipo, S. O. , Djagoun, C. A. , Nneji, I. C. , Adedeji, B. E. , Olatunde, O. , Ayoola, A. O. , Ikhimiukor, O. O. , & Okeyoyin, A. O. (2020). DNA barcoding silver butter catfish (Schilbe intermedius) reveals patterns of mitochondrial genetic diversity across african river systems. Scientific Reports, 10, 1–9. 32341417 10.1038/s41598-020-63837-4 PMC7184614

[jfb70245-bib-0061] Oliveira, L. , Knebelsberger, T. , Landi, M. , Soares, P. , Raupach, M. , & Costa, F. O. (2016). Assembling and auditing a comprehensive DNA barcode reference library for European marine fishes. Journal of Fish Biology, 89, 2741–2754. 27739061 10.1111/jfb.13169

[jfb70245-bib-0062] Ollé‐Vilanova, J. , Hajjej, G. , Macias, D. , Saber, S. , Lino, P. G. , Muñoz‐Lechuga, R. , Baibbat, S. A. , Sow, F. N. , Diaha, N. G. , Araguas, R. M. , Sanz, N. , & Vinas, J. (2024). Atlantic bonito (*Sarda sarda*) genomic analysis reveals population differentiation across Northeast Atlantic and mediterranean locations: Implications for fishery management. Marine Environmental Research, 196, 106408. 38402010 10.1016/j.marenvres.2024.106408

[jfb70245-bib-0063] Palumbi, S. R. (1997). Molecular biogeography of the Pacific. Coral Reefs, 16, S47–S52.

[jfb70245-bib-0064] Paradis, E. (2010). Pegas: An R package for population genetics with an integrated–modular approach. Bioinformatics, 26, 419–420. 20080509 10.1093/bioinformatics/btp696

[jfb70245-bib-0065] Paradis, E. , & Schliep, K. (2019). Ape 5.0: An environment for modern phylogenetics and evolutionary analyses in R. Bioinformatics, 35, 526–528. 30016406 10.1093/bioinformatics/bty633

[jfb70245-bib-0066] Patarnello, T. , Volckaert, F. A. , & Castilho, R. (2007). Pillars of Hercules: Is the Atlantic–Mediterranean transition a phylogeographical break? Molecular Ecology, 16, 4426–4444. 17908222 10.1111/j.1365-294X.2007.03477.x

[jfb70245-bib-0067] Pereira, L. , Freitas, F. , Fernandes, V. , Pereira, J. B. , Costa, M. D. , Costa, S. , Máximo, V. , Macaulay, V. , Rocha, R. , & Samuels, D. C. (2009). The diversity present in 5140 human mitochondrial genomes. The American Journal of Human Genetics, 84, 628–640. 19426953 10.1016/j.ajhg.2009.04.013 PMC2681004

[jfb70245-bib-0068] Porter, T. M. , & Hajibabaei, M. (2018). Over 2.5 million COI sequences in GenBank and growing. PLoS One, 13, e0200177. 30192752 10.1371/journal.pone.0200177 PMC6128447

[jfb70245-bib-0069] Puillandre, N. , Brouillet, S. , & Achaz, G. (2021). ASAP: Assemble species by automatic partitioning. Molecular Ecology Resources, 21, 609–620. 33058550 10.1111/1755-0998.13281

[jfb70245-bib-0070] Rabosky, D. L. , Chang, J. , Title, P. O. , Cowman, P. F. , Sallan, L. , Friedman, M. , Kaschner, K. , Garilao, C. , Near, T. J. , Coll, M. , & Alfaro, M. E. (2018). An inverse latitudinal gradient in speciation rate for marine fishes. Nature, 559, 392–395. 29973726 10.1038/s41586-018-0273-1

[jfb70245-bib-0071] Radenac, M.‐H. , Jouanno, J. , Tchamabi, C. C. , Awo, M. , Bourlès, B. , Arnault, S. , & Aumont, O. (2020). Physical drivers of the nitrate seasonal variability in the Atlantic cold tongue. Biogeosciences, 17, 529–545.

[jfb70245-bib-0093] Rambaut, A. , Drummond, A. J. , Xie, D. , Baele, G. , & Suchard, M. A. (2018). Posterior summarization in bayesian phylogenetics using tracer 1.7. Systematic Biology, 67(5), 901–904. 29718447 10.1093/sysbio/syy032 PMC6101584

[jfb70245-bib-0072] Ratnasingham, S. , & Hebert, P. D. (2013). A DNA‐based registry for all animal species: The barcode index number (BIN) system. PLoS One, 8, e66213. 23861743 10.1371/journal.pone.0066213 PMC3704603

[jfb70245-bib-0073] Ratnasingham, S. , Wei, C. , Chan, D. , Agda, J. , Agda, J. , Ballesteros‐Mejia, L. , & Hebert, P. D. N. (2024). BOLD v4: A centralized bioinformatics platform for DNA‐based biodiversity data. In R. DeSalle (Ed.), DNA barcoding: Methods and protocols (pp. 403–441). Springer US. 10.1007/978-1-0716-3581-0_26 38683334

[jfb70245-bib-0074] Reece, J. S. , Bowen, B. W. , Smith, D. G. , & Larson, A. (2010). Molecular phylogenetics of moray eels (Muraenidae) demonstrates multiple origins of a shell‐crushing jaw (Gymnomuraena, echidna) and multiple colonizations of the Atlantic Ocean. Molecular Phylogenetics and Evolution, 57, 829–835. 20674752 10.1016/j.ympev.2010.07.013

[jfb70245-bib-0075] Ruzzante, D. E. , Taggart, C. T. , Cook, D. , & Goddard, S. V. (1997). Genetic differentiation between inshore and offshore Atlantic cod (Gadus morhua) off Newfoundland: A test and evidence of temporal stability. Canadian Journal of Fisheries and Aquatic Sciences, 54, 2700–2708.

[jfb70245-bib-0076] Salmenkova, E. A. (2011). New view on the population genetic structure of marine fish. Russian Journal of Genetics, 47, 1279–1287. 22332403

[jfb70245-bib-0077] Shakouri, M. , Mostafavi, P. G. , Pourkazemi, M. , & Fatemi, S. R. (2023). Cytochrome oxidase I (COI) revealed differentiation among populations of Atule mate in the coastal waters of Persian gulf and sea of Oman (Bushehr, Bandar Abbas, Chabahar). Regional Studies in Marine Science, 60, 102833.

[jfb70245-bib-0078] Shen, K.‐N. , Chang, C.‐W. , & Durand, J.‐D. (2015). Spawning segregation and philopatry are major prezygotic barriers in sympatric cryptic Mugil cephalus species. Comptes Rendus Biologies, 338, 803–811. 26563557 10.1016/j.crvi.2015.07.009

[jfb70245-bib-0079] Shen, K.‐N. , Jamandre, B. W. , Hsu, C.‐C. , Tzeng, W.‐N. , & Durand, J.‐D. (2011). Plio‐Pleistocene sea level and temperature fluctuations in the northwestern Pacific promoted speciation in the globally‐distributed flathead mullet Mugil cephalus. BMC Evolutionary Biology, 11, 1–17. 21450111 10.1186/1471-2148-11-83 PMC3079632

[jfb70245-bib-0080] Suchard, M. A. , Lemey, P. , Baele, G. , Ayres, D. L. , Drummond, A. J. , & Rambaut, A. (2018). Bayesian phylogenetic and phylodynamic data integration using BEAST 1.10. Virus Evolution, 4, vey016. 29942656 10.1093/ve/vey016 PMC6007674

[jfb70245-bib-0081] Tamura, K. , Stecher, G. , & Kumar, S. (2021). MEGA11: Molecular evolutionary genetics analysis version 11. Molecular Biology and Evolution, 38, 3022–3027. 33892491 10.1093/molbev/msab120 PMC8233496

[jfb70245-bib-0082] Tapia, F. J. , Navarrete, S. A. , Castillo, M. , Menge, B. A. , Castilla, J. C. , Largier, J. , Wieters, E. A. , Broitman, B. L. , & Barth, J. A. (2009). Thermal indices of upwelling effects on inner‐shelf habitats. Progress in Oceanography, 83, 278–287.

[jfb70245-bib-0083] Thieme, P. , Reisser, C. , Bouvier, C. , Rieuvilleneuve, F. , Béarez, P. , Coleman, R. R. , & Durand, J.‐D. (2025). Historical biogeography of the Mugil cephalus species complex and its rapid global colonization. Molecular Phylogenetics and Evolution, 205, 108296. 39884517 10.1016/j.ympev.2025.108296

[jfb70245-bib-0084] Thompson, J. D. , Higgins, D. G. , & Gibson, T. J. (1994). CLUSTAL W: Improving the sensitivity of progressive multiple sequence alignment through sequence weighting, position‐specific gap penalties and weight matrix choice. Nucleic Acids Research, 22, 4673–4680. 7984417 10.1093/nar/22.22.4673 PMC308517

[jfb70245-bib-0085] Treml, E. A. , Roberts, J. J. , Chao, Y. , Halpin, P. N. , Possingham, H. P. , & Riginos, C. (2012). Reproductive output and duration of the pelagic larval stage determine seascape‐wide connectivity of marine populations. Integrative and Comparative Biology, 52, 525–537. 22821585 10.1093/icb/ics101

[jfb70245-bib-0086] Vilasboa, A. , Lamarca, F. , Solé‐Cava, A. M. , & Vianna, M. (2022). Genetic evidence for cryptic species in the vulnerable spiny butterfly ray Gymnura altavela (Rajiformes: Gymnuridae). Journal of the Marine Biological Association of the United Kingdom, 102, 345–349.

[jfb70245-bib-0087] Vrgoč, N. , KrstulovićŠifner, S. , Dadić, V. , & Jukić‐Peladić, S. (2006). Demographic structure and distribution of John dory, *Zeus faber* L. 1758, in the Adriatic Sea. Journal of Applied Ichthyology, 22, 205–208.

[jfb70245-bib-0088] Ward, R. D. , Costa, F. O. , Holmes, B. H. , & Steinke, D. (2008). DNA barcoding of shared fish species from the North Atlantic and Australasia: Minimal divergence for most taxa, but *Zeus faber* and *Lepidopus caudatus* each probably constitute two species. Aquatic Biology, 3, 71–78.

[jfb70245-bib-0089] Ward, R. D. , Zemlak, T. S. , Innes, B. H. , Last, P. R. , & Hebert, P. D. N. (2005). DNA barcoding Australia's fish species. Philosophical Transactions of the Royal Society of London. Series B, Biological Sciences, 360, 1847–1857. 16214743 10.1098/rstb.2005.1716 PMC1609232

[jfb70245-bib-0090] Whitfield, A. , Panfili, J. , & Durand, J. (2012). A global review of the cosmopolitan flathead mullet Mugil cephalus Linnaeus 1758 (Teleostei: Mugilidae), with emphasis on the biology, genetics, ecology and fisheries aspects of this apparent species complex. Reviews in Fish Biology and Fisheries, 22, 641–681.

[jfb70245-bib-0091] Yoneda, M. , Yamasaki, S. , Yamamoto, K. , Horikawa, H. , & Matsuyama, M. (2002). Age and growth of John dory, Zeus faber (Linnaeus, 1758), in the East China Sea. ICES Journal of Marine Science, 59, 749–756.

[jfb70245-bib-0092] Zemlak, T. S. , Ward, R. D. , Connell, A. D. , Holmes, B. H. , & Hebert, P. D. (2009). DNA barcoding reveals overlooked marine fishes. Molecular Ecology Resources, 9(s1), 237–242. 21564983 10.1111/j.1755-0998.2009.02649.x

