# Supplementary material for: Phylogeography of the widely distributed John Dory ( Zeus faber , Actinopterygii: Zeiformes) reaffirms the prevalence of at least two deeply divergent clades
Source: J Fish Biol. 2025 Oct 6;108(1):373–86. doi: 10.1111/jfb.70245 (PMC13033964; doi:10.1111/jfb.70245)
Supplement: Supplementary file 1 — Data S1. Supporting information. [file JFB-108-373-s001.docx]

**Supplementary Material 1**

**Table S1.** GenBank accession numbers, BOLD process IDs, and collection metadata for *Zeus faber* samples collected for this study. An asterisk (*) indicates samples obtained from fish markets at major landing sites, with the listed geographic coordinates representing the landing locations.

| GenBank Accession | BOLD Process ID | Country | Latitude | Longitude |
| --- | --- | --- | --- | --- |
| PX365684 | ATLMF004-21 | Portugal | 41.808 | -8.988 |
| PX365682 | ATLMF005-21 | Portugal | 41.808 | -8.988 |
| PX365652 | ATLMF006-21 | Portugal | 41.624 | -8.990 |
| PX365668 | ATLMF017-21 | Portugal | 41.490 | -9.188 |
| PX365631 | ATLMF070-21 | Portugal | 41.133 | -8.984 |
| PX365683 | ATLMF074-21 | Portugal | 40.988 | -8.996 |
| PX365646 | ATLMF090-21 | Portugal | 40.882 | -9.186 |
| PX365617 | ATLMF091-21 | Portugal | 40.882 | -9.186 |
| PX365628 | ATLMF093-21 | Portugal | 40.882 | -9.186 |
| PX365686 | ATLMF096-21 | Portugal | 40.677 | -9.210 |
| PX365622 | ATLMF097-21 | Portugal | 40.677 | -9.210 |
| PX365693 | ATLMF101-21 | Portugal | 40.677 | -9.210 |
| PX365670 | ATLMF102-21 | Portugal | 40.648 | -9.088 |
| PX365666 | ATLMF113-21 | Portugal | 40.403 | -9.120 |
| PX365654 | ATLMF114-21 | Portugal | 40.403 | -9.120 |
| PX365656 | ATLMF115-21 | Portugal | 40.539 | -9.361 |
| PX365633 | ATLMF120-21 | Portugal | 40.465 | -9.438 |
| PX365696 | ATLMF122-21 | Portugal | 40.465 | -9.438 |
| PX365619 | FDNAM071-22 | Guinea-Bissau | 12.076 | -17.138 |
| PX365624 | FDNAM072-22 | Guinea-Bissau | 12.076 | -17.138 |
| PX365685 | FDNAM073-22 | Guinea-Bissau | 12.076 | -17.138 |
| PX365648 | FDNAM074-22 | Guinea-Bissau | 12.076 | -17.138 |
| PX365678 | FDNAM075-22 | Guinea-Bissau | 12.076 | -17.138 |
| PX365621 | FDNAM284-24 | Guinea-Bissau | 11.552 | -17.173 |
| PX365671 | FDNAM285-24 | Guinea-Bissau | 11.552 | -17.173 |
| PX365663 | FDNAM286-24 | Guinea-Bissau | 11.552 | -17.173 |
| PX365647 | FDNAM287-24 | Guinea-Bissau | 11.552 | -17.173 |
| PX365665 | FDNAM288-24 | Guinea-Bissau | 11.552 | -17.173 |
| PX365636 | ZFA001-16 | Angola | -12.943 | 11.816 |
| PX365679 | ZFA002-16 | Angola | -6.867 | 12.134 |
| PX365625 | ZFA003-16 | Angola | -12.943 | 11.816 |
| PX365642 | ZFA004-16 | Angola | -7.851 | 13.002 |
| PX365629 | ZFA005-16 | Angola | -7.851 | 13.002 |
| PX365660 | ZFA006-16 | Angola | -7.851 | 13.002 |
| PX365626 | ZFA008-16 | Angola | -12.134 | 13.434 |
| PX365601 | ZFA009-16 | Angola | -12.491 | 13.348 |
| PX365687 | ZFA010-16 | Angola | -12.491 | 13.348 |
| PX365653 | ZFA011-16 | Angola | -12.491 | 13.348 |
| PX365667 | ZFA013-16 | Angola | -12.968 | 12.819 |
| PX365620 | ZFA014-16 | Angola | -12.968 | 12.819 |
| PX365659 | ZFA015-16 | Angola | -12.968 | 12.819 |
| PX365612 | ZFA020-16 | Angola | -15.352 | 11.936 |
| PX365700 | ZFA021-16 | Angola | -16.285 | 11.502 |
| PX365630 | ZFA022-16 | Angola | -16.285 | 11.502 |
| PX365681 | ZFA023-16 | Angola | -16.285 | 11.502 |
| PX365637 | ZFA024-16 | Angola | -16.285 | 11.502 |
| PX365638 | ZFA026-16 | Angola | -7.483 | 12.552 |
| PX365608 | ZFA031-16 | Angola | -7.851 | 13.002 |
| PX365697 | ZFA034-16 | Angola | -12.491 | 13.348 |
| PX365623 | ZFA036-16 | Angola | -15.155 | 12.063 |
| PX365640 | RETRO145-23 | Morocco | 25.033 | -15.768 |
| PX365672 | RETRO146-23 | Morocco | 21.778 | -17.423 |
| PX365641 | ETPQ102-23 | Morocco | 35.199 | -2.786 |
| PX365609 | ETPQ103-23 | Morocco | 35.199 | -2.786 |
| PX365689 | ETPQ345-24 | Morocco | 35.563 | -8.095 |
| PX365698 | PGFLU335-24 | Morocco | 24.048 | -16.043 |
| PX365616 | PGFLU336-24 | Morocco | 24.048 | -16.043 |
| PX365650 | PGFLU337-24 | Morocco | 29.984 | -10.087 |
| PX365614 | PGFLU338-24 | Morocco | 29.984 | -10.087 |
| PX365674 | PGFLU339-24 | Morocco | 29.984 | -10.087 |
| PX365635 | PGFLU340-24 | Morocco | 29.984 | -10.087 |
| PX365673 | PGFLU341-24 | Morocco | 29.984 | -10.087 |
| PX365658 | PGFLU342-24 | Morocco | 31.096 | -10.159 |
| PX365634 | PGFLU343-24 | Morocco | 31.332 | -10.247 |
| PX365664 | PGFLU344-24 | Morocco | 35.264 | -2.628 |
| PX365613 | PGFLU345-24 | Morocco | 35.264 | -2.628 |
| PX365606 | PGFLU405-24 | Morocco | 29.984 | -10.087 |
| PX365657 | PGFLU406-24 | Morocco | 29.984 | -10.087 |
| PX365661 | PGFLU407-24 | Morocco | 29.984 | -10.087 |
| PX365604 | PGFLU408-24 | Morocco | 29.984 | -10.087 |
| PX365691 | PGFLU409-24 | Morocco | 29.984 | -10.087 |
| PX365699 | PGFLU410-24 | Morocco | 30.526 | -9.993 |
| PX365610 | PGFLU411-24 | Morocco | 30.526 | -9.993 |
| PX365603 | PGFLU412-24 | Morocco | 30.526 | -9.993 |
| PX365688 | PGFLU413-24 | Morocco | 30.526 | -9.993 |
| PX365662 | PGFLU414-24 | Morocco | 30.526 | -9.993 |
| PX365690 | PGFLU415-24 | Morocco | 35.236 | -4.700 |
| PX365669 | PGFLU416-24 | Morocco | 22.248 | -16.874 |
| PX365639 | PGFLU453-24 | Morocco | 30.477 | -9.747 |
| PX365605 | PGFLU454-24 | Morocco | 30.477 | -9.747 |
| PX365627 | PGFLU455-24 | Morocco | 30.477 | -9.747 |
| PX365695 | PGFLU456-24 | Morocco | 30.477 | -9.747 |
| PX365680 | PGFLU457-24 | Morocco | 30.477 | -9.747 |
| PX365645 | PGFLU458-24 | Morocco | 30.477 | -9.747 |
| PX365651 | PGFLU459-24 | Morocco | 30.477 | -9.747 |
| PX365632 | PGFLU460-24 | Morocco | 30.477 | -9.747 |
| PX365602 | PGFLU461-24 | Morocco | 30.674 | -9.921 |
| PX365655 | PGFLU462-24 | Morocco | 30.674 | -9.921 |
| PX365618 | PGFLU463-24 | Morocco | 35.739 | -6.158 |
| PX365607 | BENIN062-24 | Benin | 6.13 | 2.469 |
| PX365675 | BENIN063-24 | Benin | 6.124 | 2.086 |
| PX365694 | LBR108-24 | Liberia | 4.215 | -7.610 |
| PX365649 | IMFIB305-22 | Cote d'Ivoire* | 4.747 | -6.621 |
| PX365677 | IMFIB306-22 | Cote d'Ivoire* | 4.747 | -6.621 |
| PX365611 | IMFIB189-22 | Cote d'Ivoire* | 4.747 | -6.621 |
| PX365676 | MAUBF132-24 | Mauritania* | 20.912 | -17.043 |
| PX365644 | SENEM048-23 | Senegal* | 14.717 | -17.431 |
| PX365692 | MAUBF038-24 | Mauritania* | 20.912 | -17.043 |
| PX365643 | IMFIB303-22 | Cote d'Ivoire* | 4.747 | -6.621 |
| PX365615 | IMFIB304-22 | Cote d'Ivoire* | 4.747 | -6.621 |

**Table S2.** Details for the generation of *Zeus faber* DNA barcodes.

| Samples | Number of samples | DNA extraction | Taq polymerase | Primers and thermocycling conditions | PCR confirmation | PCR product purification | Sanger sequencing |
| --- | --- | --- | --- | --- | --- | --- | --- |
| Angola | 22 | DNeasy extraction kit (Qiagen) | Speedy Supreme NzyTaq (NZYTech) | COI-3 Cocktail (Ivanova et al. 2007) | 1% agarose gel | Exo-SAP (Thermo Fisher Scientific) | External service (Macrogen Europe) |
| Morocco / Liberia / Benin | 42 | PureLink Genomic DNA Mini Kit (Invitrogen) | Platinum Taq DNA Polymerase (Invitrogen) | COI-3 Cocktail (Ivanova et al. 2007) | 2% agarose gel | ExoSAP-IT Express (Thermo Fisher Scientific) | ABI 3500 genetic analyzer (Applied Biosystems) |
| Senegal/Côte d'Ivoire / Mauritania | 8 | PureLink Genomic DNA Mini Kit (Invitrogen) | DreamTaq (Thermo Fisher) | FishF1F2 / FishR1 (Ward et al. 2005) | 1% agarose gel | External service (GenoScreen) | External service (GenoScreen) |
| Guinea-Bissau/Portugal | 28 | QuickExtract DNA Extraction Solution (Lucigen) | Speedy Supreme NzyTaq (NZYTech) | COI-2 Cocktail (Ivanova et al. 2007) | 1% agarose gel | Exo-SAP (Thermo Fisher Scientific) | External service (Macrogen Europe) |

**Table S3.** BOLD Process IDs and corresponding species names for records mined from the BOLD database. An asterisk (*) indicates samples without valid geographic coordinates but with partial geographic information in the metadata. A dagger (†) indicates samples with no geographic information in the metadata.

| BOLD Process ID | Species Name |
| --- | --- |
| ANGBF47064-19 | *Zenopsis conchifer* |
| ANGBF47065-19 | *Zenopsis conchifer* |
| ANGBF47066-19 | *Zenopsis conchifer* |
| ANGBF47068-19 | *Zenopsis conchifer* |
| ANGBF47069-19 | *Zenopsis conchifer* |
| ANGBF47070-19 | *Zenopsis conchifer* |
| FARG565-09 | *Zenopsis conchifer* |
| GBGCA13299-15 | *Zenopsis conchifer* |
| GBMIN119126-17 | *Zenopsis conchifer* |
| GBMIN119127-17 | *Zenopsis conchifer* |
| GBMIN129032-17 | *Zenopsis conchifer* |
| GBMIN129033-17 | *Zenopsis conchifer* |
| GBMIN94512-17 | *Zenopsis conchifer* |
| GBMIN94513-17 | *Zenopsis conchifer* |
| GBMNB11444-20 | *Zenopsis conchifer* |
| HVDBF495-12 | *Zenopsis conchifer* |
| HVDBF496-12 | *Zenopsis conchifer* |
| HVDBF497-12 | *Zenopsis conchifer* |
| SCAFB131-07 | *Zenopsis conchifer* |
| SCFAC757-06 | *Zenopsis conchifer* |
| SCFAC764-06 | *Zenopsis conchifer* |
| ABFJ126-06 | *Zenopsis nebulosa* |
| FMVIC427-08 | *Zenopsis nebulosa* |
| FMVIC923-08 | *Zenopsis nebulosa* |
| FMVIC924-08 | *Zenopsis nebulosa* |
| FNZ874-07 | *Zenopsis nebulosa* |
| FOAD433-05 | *Zenopsis nebulosa* |
| FOAD434-05 | *Zenopsis nebulosa* |
| FOAD435-05 | *Zenopsis nebulosa* |
| FTW871-09 | *Zenopsis nebulosa* |
| GBGCA4395-13 | *Zenopsis nebulosa* |
| GBGCA4556-13 | *Zenopsis nebulosa* |
| PHILA1782-16 | *Zenopsis nebulosa* |
| ANGBF7100-12 | *Zeus capensis* |
| ANGBF7101-12 | *Zeus capensis* |
| ANGBF7191-12 | *Zeus capensis* |
| DSFSE140-07 | *Zeus capensis* |
| DSFSE141-07 | *Zeus capensis* |
| DSFSE142-07 | *Zeus capensis* |
| DSFSE143-07 | *Zeus capensis* |
| TZMSB306-04 | *Zeus capensis* |
| TZMSB307-04 | *Zeus capensis* |
| TZMSB308-04 | *Zeus capensis* |
| GBGC1138-06 | *Zeus faber** |
| FMVIC428-08 | *Zeus faber* |
| FOAD443-05 | *Zeus faber* |
| FOAD444-05 | *Zeus faber* |
| FOAD445-05 | *Zeus faber* |
| FOAD446-05 | *Zeus faber* |
| FOAD447-05 | *Zeus faber* |
| FOAF176-07 | *Zeus faber* |
| FOAK598-10 | *Zeus faber* |
| FOAN1099-11 | *Zeus faber* |
| SDP331037-16 | *Zeus faber** |
| SDP331087-16 | *Zeus faber** |
| GBGCA4392-13 | *Zeus faber** |
| GBGCA4394-13 | *Zeus faber** |
| GBGCA4537-13 | *Zeus faber** |
| ZOSKT1581-16 | *Zeus faber** |
| ZOSKT2080-16 | *Zeus faber** |
| GBMIN130984-17 | *Zeus faber*† |
| GBMIN130985-17 | *Zeus faber*† |
| GBMIN130986-17 | *Zeus faber*† |
| GBMIN130987-17 | *Zeus faber*† |
| GBMIN130988-17 | *Zeus faber*† |
| ANGBF57412-22 | *Zeus faber** |
| ANGBF9480-12 | *Zeus faber** |
| ANGBF9481-12 | *Zeus faber** |
| ANGBF9713-12 | *Zeus faber** |
| ANGBF9714-12 | *Zeus faber** |
| ANGBF9479-12 | *Zeus faber** |
| ANGBF9709-12 | *Zeus faber** |
| ANGBF9712-12 | *Zeus faber** |
| ANGBF9851-12 | *Zeus faber** |
| BIM416-15 | *Zeus faber* |
| BIM726-19 | *Zeus faber* |
| ANGBF9637-12 | *Zeus faber** |
| ANGBF9660-12 | *Zeus faber** |
| CSFOM087-10 | *Zeus faber** |
| ANGBF50351-19 | *Zeus faber** |
| FMVIC926-08 | *Zeus faber* |
| FCFMT001-07 | *Zeus faber* |
| FCFMT005-07 | *Zeus faber* |
| FCFMT036-07 | *Zeus faber* |
| FCFMT056-07 | *Zeus faber* |
| FCFMT062-07 | *Zeus faber* |
| FNZA391-08 | *Zeus faber** |
| FNZA392-08 | *Zeus faber** |
| FNZA393-08 | *Zeus faber** |
| FNZA394-08 | *Zeus faber** |
| FNZA395-08 | *Zeus faber** |
| NOMFO004-17 | *Zeus faber* |
| PHILA1790-16 | *Zeus faber* |
| PHILA1858-16 | *Zeus faber* |
| FCFP079-05 | *Zeus faber* |
| FCFP080-05 | *Zeus faber* |
| FCFP152-05 | *Zeus faber* |
| FCFP153-05 | *Zeus faber* |
| FCFP154-05 | *Zeus faber* |
| FCFPS047-06 | *Zeus faber** |
| FCFPS048-06 | *Zeus faber** |
| FCFPW011-06 | *Zeus faber* |
| FCFPW012-06 | *Zeus faber* |
| FCFPW013-06 | *Zeus faber* |
| FCFPW014-06 | *Zeus faber* |
| FCFPW028-06 | *Zeus faber* |
| FCFPW100-06 | *Zeus faber* |
| FCFPW101-06 | *Zeus faber* |
| FCFPW102-06 | *Zeus faber* |
| MLFPI171-10 | *Zeus faber* |
| MLFPI172-10 | *Zeus faber* |
| MLFPI359-14 | *Zeus faber* |
| MLFPI360-14 | *Zeus faber* |
| MLFPI361-14 | *Zeus faber* |
| MLFPI362-14 | *Zeus faber* |
| GBMTG235-16 | *Zeus faber*† |
| DSFSG094-10 | *Zeus faber* |
| DSFSG778-12 | *Zeus faber* |
| TZMSC208-05 | *Zeus faber* |
| TZSAL128-04 | *Zeus faber* |
| ANGBF9476-12 | *Zeus faber** |
| ANGBF9477-12 | *Zeus faber** |
| ANGBF9710-12 | *Zeus faber** |
| GBGCA8533-15 | *Zeus faber* |
| GBGCA9620-15 | *Zeus faber* |
| FTW628-09 | *Zeus faber* |
| FTW629-09 | *Zeus faber* |
| ZOSKT2081-16 | *Zeus faber** |
| ANGBF47071-19 | *Zeus faber** |
| ANGBF9478-12 | *Zeus faber** |
| ANGBF9711-12 | *Zeus faber** |
| GBMIN121596-17 | *Zeus faber** |
| GBMIN126293-17 | *Zeus faber** |
| GBMIN132008-17 | *Zeus faber** |
| BNSF247-11 | *Zeus faber* |
| BNSF248-11 | *Zeus faber* |
| BNSFI051-12 | *Zeus faber* |
| BNSFI052-12 | *Zeus faber* |
| BNSFI053-12 | *Zeus faber* |
| FCFUK007-06 | *Zeus faber* |
| FCFUK037-06 | *Zeus faber* |
| FCFUK051-06 | *Zeus faber* |
| RFE383-05 | *Zeus faber*† |
| RFE384-05 | *Zeus faber*† |

**Table S4.** Summary of Kimura 2-Parameter (K2P) distance metrics for the *Zeus faber* dataset and for each of the two main clades, based on both the reconstructed 620 bp and non-reconstructed 408 bp multiple sequence alignments. Mean K2P distances are provided with standard error (SE).

| K2P distance (%) | Non-reconstructed alignment (408bp) | Reconstructed alignment (620bp) |
| --- | --- | --- |
| Mean Overall ± SE | 4.7±0.03 | 3.9±0.024 |
| Max. Overall | 10.9 | 8.8 |
| Mean Clade A ± SE | 0.1±0.002 | 0.2±0.002 |
| Max. Clade A | 0.7 | 0.8 |
| Mean Clade B ± SE | 1.1 ± 0.013 | 0.9±0.011 |
| Max. Clade B | 2.8 | 2.3 |
| Mean Between Clades ± SE | 9.0±0.003 | 7.4±0.003 |

**Table S5.** Genetic diversity indices for the entire *Zeus faber* dataset and for each of the two main clades, based on the non-reconstructed 408 bp multiple sequence alignment. N – number of individuals; S – number of polymorphic sites; PIS – number of parsimony-informative sites; H – number of haplotypes; Hd – haplotype diversity; π – nucleotide diversity.

|  | Clade A | Clade B | Overall |
| --- | --- | --- | --- |
| N | 112 | 89 | 201 |
| S | 8 | 24 | 53 |
| PIS | 3 | 14 | 42 |
| H | 9 | 16 | 25 |
| Hd | 0.384 | 0.753 | 0.761 |
| π | 0.0011 | 0.0106 | 0.0434 |

**Table S6.** Mean Kimura-2-Parameter (K2P) distance values (% ± standard error) between geographic groups of *Zeus faber* based on the non-reconstructed 408 bp multiple sequence alignment.

|  | Mediterranean | Australia / New Zealand | Northeast Atlantic | Asia | Atlantic Africa | South Africa |
| --- | --- | --- | --- | --- | --- | --- |
| Mediterranean | – |  |  |  |  |  |
| Australia / New Zealand | 9.2±0.01 | – |  |  |  |  |
| Northeast Atlantic | 0.1±0.004 | 9.1±0.005 | – |  |  |  |
| Asia | 9.5±0.019 | 1.4±0.023 | 9.4±0.01 | – |  |  |
| Atlantic Africa | 8.9±0.006 | 1.6±0.008 | 8.8±0.003 | 1.9±0.012 | – |  |
| South Africa | 8.9±0.02 | 0.8±0.024 | 8.8±0.007 | 1.1±0.032 | 1.8±0.008 | – |

**Table S7.** Number of samples, genetic diversity indices, and Tajima’s D test for the geographic groups in the *Zeus faber* dataset based on the non-reconstructed 408bp alignment. N – number of individuals; S – number of polymorphic sites; PIS – number of parsimony-informative sites; H – number of haplotypes; Hd – haplotype diversity; π – nucleotide diversity; D – Tajima’s D; p-value – Tajima’s D p-value.

| Raw data | N | S | PIS | H | Hd | π | D | D’s p-value |
| --- | --- | --- | --- | --- | --- | --- | --- | --- |
| Mediterranean Sea | 26 | 3 | 1 | 4 | 0.483 | 0.0015 | -0.591 | 0.596 |
| Northeast Atlantic Ocean | 86 | 6 | 3 | 7 | 0.349 | 0.0009 | -1.574 | 0.096 |
| Atlantic Africa | 48 | 5 | 2 | 6 | 0.337 | 0.0009 | -1.658 | 0.078 |
| Australia / New Zealand | 16 | 9 | 0 | 4 | 0.350 | 0.0028 | -2.149 | 0.008 |
| Asia | 12 | 6 | 1 | 4 | 0.636 | 0.0030 | -1.429 | 0.151 |
| South Africa | 4 | *–* | *–* | 1 | *–* | *–* | *–* | *–* |
| Subsampled data | **N** | **S** | **PIS** | **H** | **Hd** | **π** | **D** | **D’s p-value** |
| Mediterranean Sea | 12 | 1.9 | 1 | 2.9 | 0.485 | 0.0015 | -0.096 | 0.714 |
| Northeast Atlantic Ocean | 12 | 1.9 | 0.4 | 2.9 | 0.370 | 0.0010 | -1.095 | 0.321 |
| Atlantic Africa | 12 | 1.9 | 0.3 | 2.9 | 0.357 | 0.0010 | -1.180 | 0.281 |
| Australia / New Zealand | 12 | 2.2 | 0 | 3.2 | 0.574 | 0.0045 | -0.703 | 0.920 |
| Asia | 12 | 6 | 1 | 4 | 0.636 | 0.0030 | -1.429 | 0.151 |
| South Africa | *–* | *–* | *–* | *–* | *–* | *–* | *–* | *–* |

**Table S8.** AMOVA results for the *Zeus faber* dataset based on the non-reconstructed 408 bp multiple sequence alignment, analysed at two hierarchical levels: among clades and among geographic groups (Northeast Atlantic, Mediterranean, Atlantic Africa, South Africa, Australia and New Zealand, and Asia).

| Source of variation | df | Sum of squares | Variance components | Percentage of variation (%) | p-value |
| --- | --- | --- | --- | --- | --- |
| Among clades | 1 | 1606.7 | 16.3 | 89.6 | 0.067 |
| Among geographic groups | 4 | 141.4 | 1.6 | 9.0 | <0.0001 |
| Within geographic groups | 186 | 47.6 | 0.3 | 1.4 | <0.0001 |

**Supplementary Text S1.** Rationale for the exclusion of a *Zeus faber* sequence downloaded from BOLD from analyses involving geographic groups.

A sequence labelled as having been collected in the Mid-Atlantic Bight (BOLD process ID: GBGC1138-06) was excluded from the analyses utilizing the established geographic groups (see Materials and Methods section), since *Zeus faber'*s distribution is not typically reported to extend to this region. While one record of *Zeus faber* in this region was found in the Global Biodiversity Information Facility (GBIF) database (iNaturalist contributors, 2025; <https://www.gbif.org/occurrence/1944391250>), we found no other evidence of the species' distribution extending into the Western Atlantic Ocean. Additionally, the images associated with the record clearly depict a specimen from the *Zenopsis* genus rather than *Zeus faber*. This conclusion was supported by morphological features, such as the presence of six large bony plates along the base of the dorsal fin and more than 23 soft rays in the anal fin. While we cannot definitively link this misidentification to the sequence in question, it is possible that an error occurred at some point during the specimen collection or data processing. Clarification or rectification of this record would be required to confirm the actual origin of the sequence. Due to the uncertainty of the record's geographic origin, we excluded it from the geographic groups' analyses, as we did with sequences where information on geography was absent.

**References**

Ivanova NV, Zemlak TS, Hanner RH, Hebert PD (2007) Universal primer cocktails for fish DNA barcoding. Molecular Ecology Notes 7:544–548. <https://doi.org/10.1111/j.1471-8286.2007.01748.x>

Ward RD, Zemlak TS, Innes BH, Last PR, Hebert PDN (2005) DNA barcoding Australia’s fish species. Philos Trans R Soc Lond B Biol Sci 360:1847–1857. <https://doi.org/10.1098/rstb.2005.1716>

iNaturalist contributors, iNaturalist (2025). iNaturalist Research-grade Observations. iNaturalist.org. Occurrence dataset <https://doi.org/10.15468/ab3s5x> accessed via GBIF.org on 2025-02-12. <https://www.gbif.org/occurrence/1944391250>
